# Supplementary material for: Source sector and fuel contributions to ambient PM2.5 and attributable mortality across multiple spatial scales
Source: Nat Commun. 2021 Jun 14;12:3594. doi: 10.1038/s41467-021-23853-y (PMC8203641; doi:10.1038/s41467-021-23853-y)
Supplement: Supplementary file 1 — Supplementary Information [file 41467_2021_23853_MOESM1_ESM.pdf]

Supplementary Information for

Source Sector and Fuel Contributions to Ambient $PM_{2.5}$ and Attributable Mortality Across Multiple Spatial Scales

Erin E. McDuffie[1,2*], Randall V. Martin[1,2], Joseph V. Spadaro[3], Richard Burnett[4], Steven J. Smith[5], Patrick O'Rourke[5], Melanie Hammer[1,2], Aaron van Donkelaar[2,1], Liam Bindle[1,2], Viral Shah[6†], Lyatt Jaeglé[6], Gan Luo[7], Fangqun Yu[7], Jamiu Adeniran[8], Jintai Lin[8], Michael Brauer[9,4]

[1]Department of Energy, Environmental, and Chemical Engineering, Washington University in St. Louis, St. Louis, MO, USA
[2]Department of Physics and Atmospheric Science, Dalhousie University, Halifax, NS, Canada
[3]Spadaro Environmental Research Consultants (SERC)
[4]Institute for Health Metrics and Evaluation, University of Washington, Seattle, WA, USA
[5]Joint Global Change Research Institute, Pacific Northwest National Laboratory, College Park, MD, USA
[6]Department of Atmospheric Sciences, University of Washington, Seattle, WA, USA
[7]Atmospheric Sciences Research Center, University at Albany, Albany, New York, USA
[8]Laboratory for Climate and Ocean-Atmosphere Studies, School of Physics, Peking University, Beijing, China
[9]School of Population and Public Health, University of British Columbia, Vancouver, BC, Canada
[†]Now at: Harvard John A. Paulson School of Engineering and Applied Sciences, Harvard University, Cambridge, MA, USA

Corresponding Author: Erin E. McDuffie
Email: erin.mcduffie@wustl.edu

This file includes:

Supplementary Text 1 to 10
Supplementary Figures 1 to 10
Supplementary Tables 1 to 2

Other Supplementary Information for this manuscript include:

Supplementary Data 1-3

Additional Supporting Tables and Figures

This supplementary information file describes the details of the analysis configuration. A key objective of including these details is to promote transparency and reproducibility. In this work, world countries and regions follow those used in the Global Burden of Disease (GBD) Project, as shown in Supplementary Table 1.

Supplementary Table 1. Member countries and territories of the 21 GBD world regions.

GBD Region	Member Countries			
**Asia Pacific, High Income**	Brunei Darussalam	Japan	South Korea	Singapore
**Asia, Central**	Armenia	Azerbaijan	Georgia	Kazakhstan
	Kyrgyzstan	Mongolia	Tajikistan	Turkmenistan
	Uzbekistan			
**Asia, East**	China	Taiwan (Province of China)		
	North Korea (Democratic People's Republic of Korea)			
**Asia, South**	Bangladesh	Bhutan	India	Nepal
	Pakistan			
**Asia, Southeast**	Cambodia	Indonesia	Malaysia	Maldives
	Mauritius	Myanmar	Philippines	Sri Lanka
	Seychelles	Thailand	Timor-Leste	Vietnam
	Laos (Loa People's Democratic Republic)			
**Oceania**	American Samoa	Cook Islands	Fiji	Guam
	Kiribati	Marshall Islands	Nauru	Niue
	Palau	Papua New Guinea	Samoa	Solomon Islands
	Tokelau	Tonga	Tuvalu	Vanuatu
	Federated States of Micronesia		Northern Mariana Islands	
**Australasia**	Australia	New Zealand		
**Caribbean**	The Bahamas	Barbados	Belize	Bermuda
	Cuba	Dominica	Dominican Republic	Grenada
	Guyana	Haiti	Jamaica	Puerto Rico
	Saint Lucia	Suriname	Antigua and Barbuda	Saint Kitts and Nevis
	Trinidad and Tobago	US Virgin Islands	Saint Vincent and the Grenadines	
**Europe, Central**	Albania	Bulgaria	Croatia	Czech Republic (Czechia)
	Hungary	North Macedonia	Montenegro	Poland
	Romania	Serbia	Slovakia	Slovenia
	Bosnia and Herzegovina			
**Europe, Eastern**	Belarus	Estonia	Latvia	Lithuania
	Republic of Moldova	Russian Federation	Ukraine	
**Europe, Western**	Andorra	Austria	Belgium	Cyprus
	Denmark	Finland	France	Germany
	Greece	Iceland	Ireland	Israel
	Italy	Luxembourg	Malta	Monaco
	Netherlands	Norway	Portugal	San Marino
	Spain	Sweden	Switzerland	United Kingdom
**North America, High Income**	Canada	Greenland	United States	
**Latin America, Andean**	Bolivia (Plurinational State of)		Ecuador	Peru
**Latin America, Central**	Colombia	Costa Rica	El Salvador	Guatemala
	Honduras	Mexico	Nicaragua	Panama
	Venezuela (Bolivarian Republic of)			
**Latin America, Southern**	Argentina	Chile	Uruguay	
**Latin America, Tropical**	Brazil	Paraguay		
**North Africa / Middle East**	Afghanistan	Algeria	Bahrain	Egypt
	Iraq	Jordan	Kuwait	Lebanon
	Libya	Morocco	Palestine	Oman
	Qatar	Saudi Arabia	Sudan	Syrian Arab Republic
	Tunisia	Turkey	Yemen	United Arab Emirates
	Iran (Islamic Republic of)			

**Sub-Saharan Africa, Central**	Angola	Congo	Equatorial Guinea	Gabon
	Central African Republic	Democratic Republic of the Congo		
**Sub-Saharan Africa, Eastern**	Burundi	Comoros	Djibouti	Eritrea
	Ethiopia	Kenya	Madagascar	Malawi
	Mozambique	Rwanda	Somalia	South Sudan
	Uganda	Zambia	United Republic of Tanzania	
**Sub-Saharan Africa, South**	Botswana	Lesotho	Namibia	South Africa
	Eswatini	Zimbabwe		
**Sub-Saharan Africa, Western**	Benin	Burkina Faso	Cameroon	Cape Verde
	Chad	Cote d'Ivoire	The Gambia	Ghana
	Guinea	Guinea-Bissau	Liberia	Mali
	Mauritania	Niger	Nigeria	Senegal
	Sierra Leone	Togo	Sao Tome and Principe	

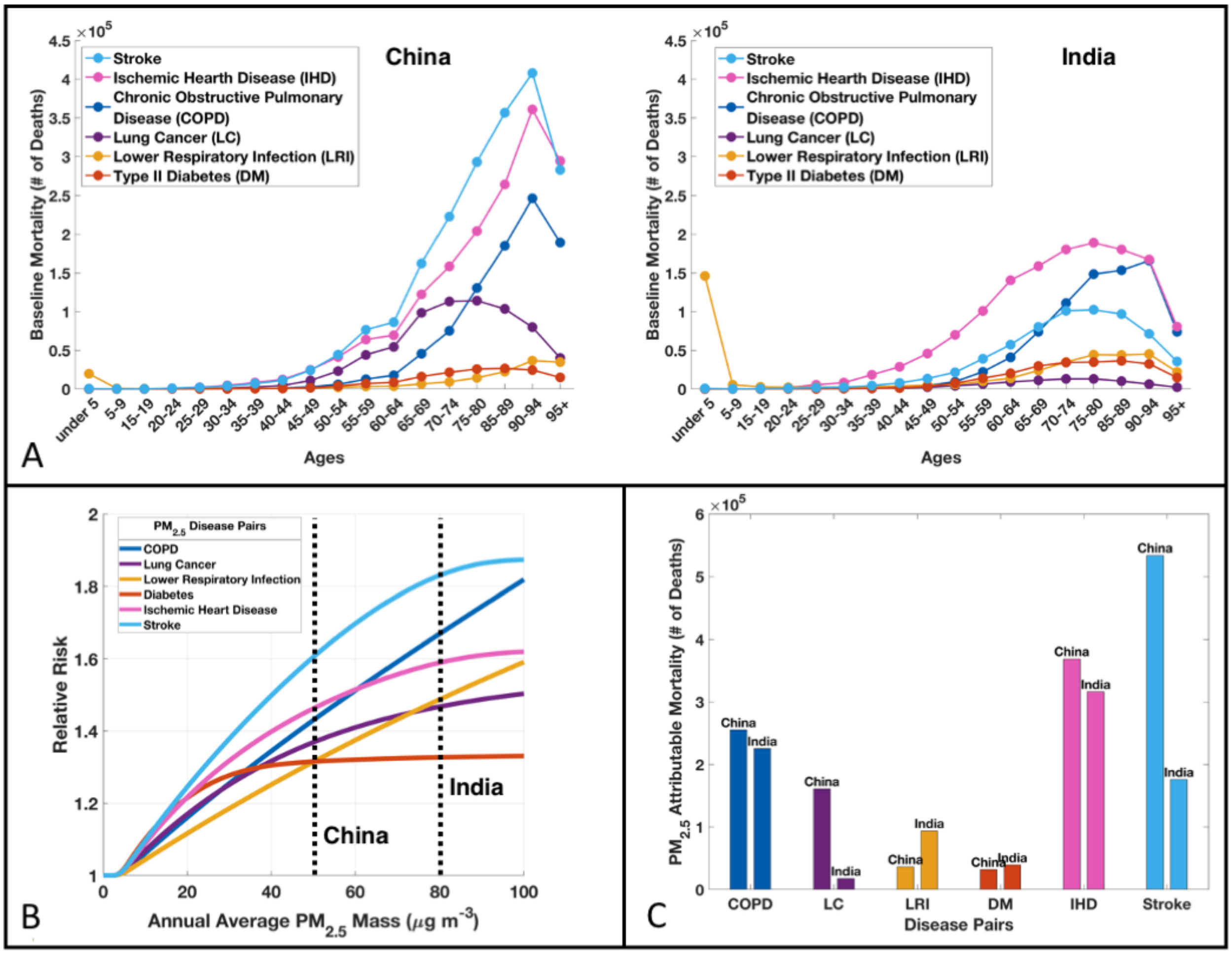

Supplementary Figure 1. $PM_{2.5}$ Disease Burden Comparison for India and China. (A) GBD2019 background baseline mortality data as a function of population age and disease. **(B)** GBD2019 concentration response functions (same as Supplementary Figure 2), with dashed lines showing population weighted mean $PM_{2.5}$ concentrations for China and India. **(C)** Total $PM_{2.5}$ attributable deaths in China and India as a function of disease.

Supplementary Text 1. 2019 Exposure Estimates – Additional Details

Following the downscaling procedure described in the Methods (and Supplementary Text 9), we apply high-resolution (gridded at ~1 km × ~1 km) exposure estimates for the year 2019 (weighted by 2019 gridded population[1]) to the GBD2019 CRFs with 2019 baseline mortality data to assess changes in the estimated disease burden between 2017 and 2019. Disease burden estimates are independent from model emission sensitivity simulations and do not require changes or projections in emissions. In both years, the same nine countries were estimated to have largest number of $PM_{2.5}$ attributable deaths, though the annual number of deaths in each country was larger in 2019 than 2017, except for in Russia. Similarly, annual population-weighted mean (PWM) $PM_{2.5}$ concentrations also increased in each of these top nine countries, except for in China and the United States. The complex relationship between annual national $PM_{2.5}$ concentrations and resulting attributable deaths highlights the importance of multiple factors in disease burden estimations. For neonatal disorders, the incidence associated with outdoor $PM_{2.5}$ exposure totaled to 2.07 (95% CI: 0.02-5.02) million worldwide, which increased marginally to 2.09 (95% CI: 0.02-5.06) million in 2019. At the sub-national level, the top four of the 200 select areas with the highest $PM_{2.5}$ concentrations (Singrauli, Kanpur, Sitapur, and Ahmedabad, India) all experienced increases in PWM $PM_{2.5}$ mass, persisting at levels between 14 and 16 times greater than the WHO annual average guidelines. Of the 200 sub-national areas, 45% experienced no change or an increase in PWM $PM_{2.5}$ concentration between 2017 and 2019. The area surrounding Pune, India had the largest absolute increase from 57 to 63.2 $\mu g\ m^{-3}$, while the area surrounding Xingping, China had the largest absolute decrease from 68.4 to 60.1 $\mu g\ m^{-3}$. These changes serve to identify potential locations with effective mitigation strategies or those locations with the most to gain from pollution reductions. Supplementary Data 3 provides the PWM values for the global area, each of the 21 world regions, 204 countries, and 200 sub-national areas.

Supplementary Text 2. GEMM Sensitivity Study – Additional Details

As an alternative to the GBD estimates, previous global studies have used the GEMM. The GEMM exclusively incorporates risk information from cohort studies of outdoor air pollution (41 cohorts from 16 countries)[2] on non-accidental mortality and was highly sensitive to one particular cohort of Chinese men[3] in the original version. Another feature of the GEMM is that

its non-accidental mortality estimate suggests a larger impact of $PM_{2.5}$ exposure on mortality than the sum of cause-specific attributable mortality estimates. As new evidence on links between $PM_{2.5}$ and other (e.g., chronic kidney disease, dementia) causes of death emerge, this difference between cause-specific and all-cause (non-accidental) attributable mortality will decrease.

Application of the GEMM-based disease-specific estimates to the disease burden, however, should also be employed with caution. As further discussed in Hystad, et al. [4] the GEMM is based on analyses of non-accidental mortality primarily derived from epidemiologic studies conducted in high-income countries. When applied to disease burden estimates, this assumes similar distributions of causes of death, including the relative proportion of the specific diseases linked to air pollution and the population age distribution in high-income countries as in low and middle-income countries. This leads to uncertainty since the relative frequencies of the various causes of deaths differ markedly between countries of the world. In particular, application to Africa and South Asia is likely to lead to substantial uncertainty. As the GBD2019 CRFs are derived directly from studies of specific diseases, they can be more reliably applied to disease-specific mortality rates across countries. Other sources of uncertainty such as the assumptions of equitoxicity, variation in e.g., healthcare access and quality and population characteristics, and extrapolation to concentrations beyond those included in epidemiologic studies are common to the application of both the GBD2019 CRFs and the GEMM.

To address some of these uncertainties, the GEMM CRFs in this work were updated as described in the Methods to include the same ambient PM cohort studies that are inputs to the GBD2019 CRFs and to add functions for diabetes and reproductive outcomes. Supplementary Figure 2 compares the disease-specific CRFs for the GBD2019 and updated GEMM. With these updates, and when restricted to the five NCDs +LRI that are included in the GBD2019 CRFs, 2017 global GEMM attributable mortality estimates were lower than previous GEMM estimates for 2015 when only the four NCDs +LRI were included (6.2 million vs 6.9 million)[2]. Globally, the updated GEMM CRFs estimated a $PM_{2.5}$ attributable burden of 6.2 (95% CI: 4.4 to 7.8) million deaths in 2017. While satellite-based estimates have shown a recent decreasing trend in PWM $PM_{2.5}$ mass across East Asia, Europe, and the Eastern U.S.[5], a detailed analysis of differences (temporal trends vs. methodological differences) between the original and updated GEMM estimates is outside the scope of this work.

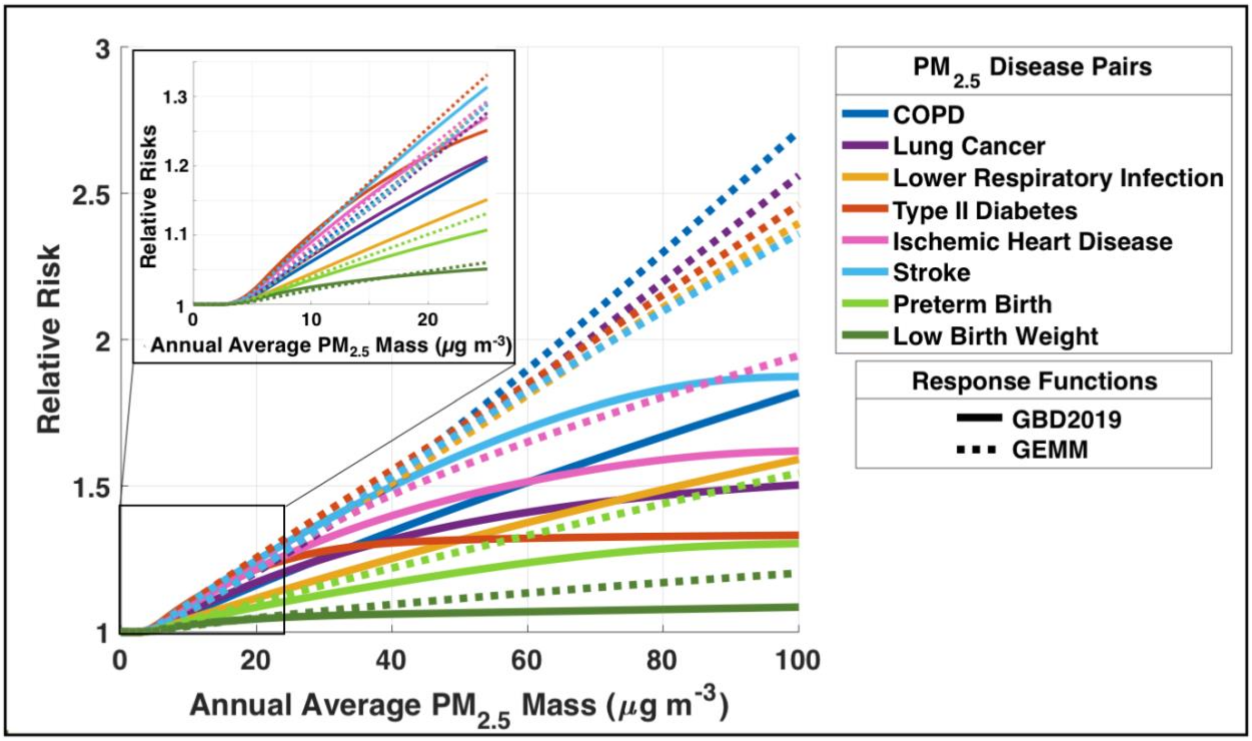

Supplementary Figure 2. Concentration response functions for the GBD2019 and updated GEMM. **Solid Lines**: GBD2019 CRFs, **Dashed Lines**: updated GEMM. Line colors correspond to the central values of eight disease-response pairs. For illustrative purposes, response curves for IHD and Stroke correspond to the 60-64 age group, COPD, LC, and DM responses are for all ages over 25 years, and LRIs are for ages under 5 years and greater than 25 years. Preterm births are at a gestational age less than 37 weeks (PTB) and weights below 2.5 kg (LBW). For illustrative purposes, the insert highlights the relative risks at exposure levels less than 25 μg m^{-3}.

Supplementary Figure 3 shows the relative disease-specific contributions from both the updated GEMM and GBD2019 CFRs for 21 world regions (A, C), and the top 20 countries with the largest number of attributable deaths (B, D). S1-Fig. 2 shows that the relative disease contributions predicted by the updated GEMM were similar to those from the GBD2019 CRFs. For example, both predict that the largest number of attributable deaths at the global scale were from IHD, and then in decreasing order from Stroke, COPD, LRI, LC, and DM. The absolute number of attributable deaths, however, were nearly always larger from the GEMM (Supplementary Data 1). Two exceptions were for North America and Australasia. These regions had the lowest PWM $PM_{2.5}$ concentrations and the difference in relative predictions reflect the lower relative risks for IHD, Stroke, and Diabetes in the updated GEMM at $PM_{2.5}$ concentrations below 10 μg m^{-3} (Supplementary Figure 2). As a result of these and other differences in the CRFs, the GEMM and GBD2019 CRFs also predicted different relative

rankings of the top 20 countries. Supplementary Figure 3b and d show that both the GBD2019 CRFs and GEMM predicted the same top two countries (China and India), but that the relative rankings for the next 18 countries differ. In addition to ranking differences, the GBD2019 CRFs also included Thailand (16th), Poland (19th), and Germany (20th) in the top 20 countries, while the GEMM alternatively predicted that Myanmar (12th), North Korea (17th), and Nepal (20th) ranked in the top 20. This comparison closes a portion of the gap between previous GEMM and GBD disease burden results, however, the accuracy of both estimates are dependent on the availability of robust and high-resolution exposure data, particularly in high exposure areas.

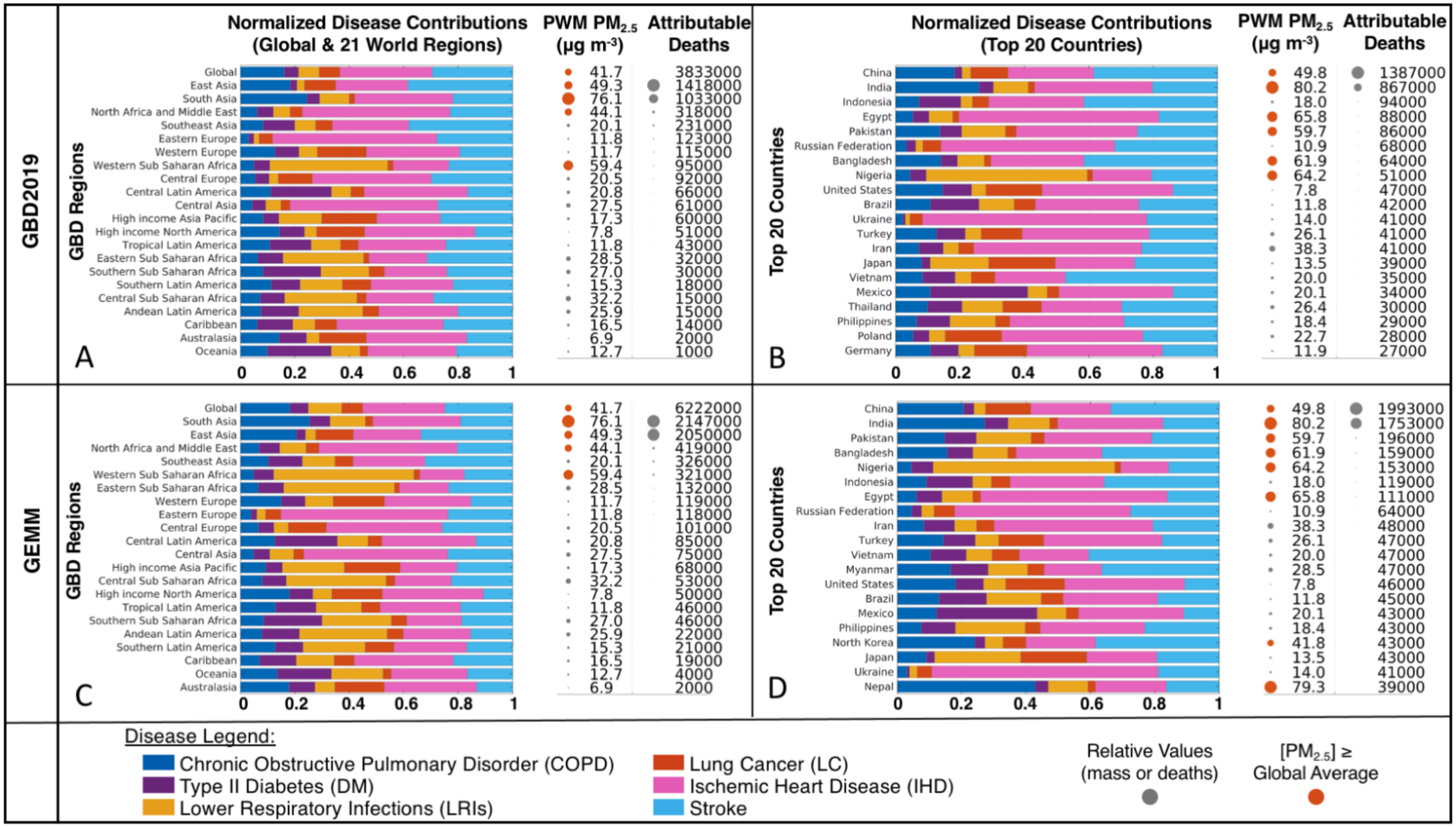

Supplementary Figure 3: Normalized disease contributions to total attributable mortality in 2017 for 21 world regions (A, C) and 20 countries (B, D) with the highest outdoor $PM_{2.5}$ disease burden. Panels show results estimated using the GBD2019 CRFs (A, B) and the updated GEMM (C, D). Bar charts show the relative contributions of six $PM_{2.5}$-disease pairs to regional and national-level outdoor $PM_{2.5}$ attributable deaths, sorted by decreasing number of deaths. The number of LBW and PTB incidences are included in Supplementary Data 1. PWM $PM_{2.5}$ concentrations and number of attributable deaths are additionally provided for each region/country. Relative amounts are illustrated by relative dot sizes (except for the global total disease burden). Red dots indicate regions/countries with $PM_{2.5}$ exposure levels equivalent or larger than the global average.

Supplementary Text 3. Global Model Details

$PM_{2.5}$ source sensitivity simulations for the year 2017 are shown in Supplementary Table 2 and are conducted with the GEOS-Chem 3D atmospheric chemical transport model[6]. The GEOS-Chem model solves for the evolution of atmospheric aerosols and gases using meteorological data, global and regional emission inventories, and algorithms that represent the physics and chemistry of atmospheric processes. Global simulations are conducted from December 2016 to January 2018 (1-month spin-up) at 2°×2.5° horizontal resolution and 47 vertical layers. Global simulations are supplemented with three additional one-way nested simulations at 0.5°×0.625° horizontal resolution that cover North America (10° N – 70° N, 140° W – 40° W), Europe (30° N – 70° N, 30° W – 50° E), and China and Southeast Asia (11° S – 55° N, 60° E – 150° E)[7]. Each simulation is driven by assimilated meteorological data from the Goddard Earth Observing System from the NASA Global Modeling and Assimilation Office (GMAO). We use the MERRA-2 historical reanalysis product, archived at a 3-hour temporal resolution for 3D fields and 1-hour for 2D fields. The transport and chemistry timesteps are set to 10 and 20 minutes respectively, to optimize simulation accuracy and computational efficiency[8].

In this work, we use the GEOS-Chem 'tropchem' chemical mechanism that includes coupled aerosol-oxidant chemistry in the troposphere and stratosphere. The gas-phase mechanism includes detailed HOx-NOx-VOC-ozone chemistry[6], coupled to aerosol chemistry for inorganic sulfate-nitrate-ammonium aerosol[9,10], as well as carbonaceous (black and organic carbon) aerosol[10,11], sea salt[12], and dust[13,14]. Relative humidity dependent aerosol size distributions and optical properties are based on the Global Aerosol Data Set[15,16], with updates for organics and secondary inorganics from observations[17,18], mineral dust[14,19,20], and absorbing brown carbon[21]. Aerosol thermodynamic partitioning between sulfate-nitrate-ammonium is computed with the ISORORPIA II thermodynamic model[22], while the BC mechanism is described by Wang, et al. [10]. We use the simple, irreversible, direct yield scheme for secondary organic aerosol (SOA) from Kim, et al. [11], as this mechanism has been shown to better reproduce available observations of global organic aerosol mass relative to the more complex scheme[23]. For physical processes, GEOS-Chem uses the TPCORE advection algorithm[24] and computes convective transport from the convective mass fluxes in the meteorological data, as described by Wu, et al. [25]. In this work, boundary layer mixing uses the non-local mixing scheme as implemented by Lin and McElroy [26].

The core source code for this work is GEOS-Chem v12.1.0[27], released Nov. 2018. To correct a long-standing bias in nitrate aerosol concentrations[28,29], the v12.1.0 source code has been updated here as part of the Global Burden of Disease – Major Air Pollution Sources project (https://sites.wustl.edu/acag/datasets/gbd-maps/). The code is available on GitHub: https://github.com/emcduffie/GC_v12.1.0_EEM. Major updates follow literature recommendations and include an updated parameterization for the heterogeneous uptake of N_2O_5 from McDuffie, et al. [30], the added heterogeneous production of $ClNO_2$ following Shah, et al. [31] and recommended $ClNO_2$ yield reductions from McDuffie, et al. [32], a reduction in the deposition of HNO_3 under cold conditions following Shah, et al. [31], as well as an update to the wet deposition scheme following recommendations in Luo, et al. [29]. In addition to the updates described in Luo, et al. [29], the rate of SO_2 removal in clouds is also reduced, and the rainout efficiencies for hydrophilic OC and BC species are reduced by 50% following recent recommendations[33]. These and additional minor code updates are described in the GitHub README file.

To evaluate the impact of these model updates, Supplementary Figure 5b shows a bar chart of the normalized mean bias (NMB) in the simulated global annual averages of aerosol nitrate, sulfate, ammonium, total organic carbon, black carbon, fine dust, and sea salt in the default v12.1.0 GEOS-Chem source code and the updated model, compared to annual average observations (described in Supplementary Text 4). As shown in Supplementary Figure 5b, the mechanistic model updates described above reduce the NMB in the updated GEOS-Chem simulated concentrations of aerosol nitrate from 2 $\mu g/m^3$ to 0.5 $\mu g/m^3$ relative to observed values. Model updates additionally improve the model-observation agreement of ammonium, black carbon, and dust compared to the default model. In contrast, the negative bias in sea salt is enhanced in the updated base simulation relative to the default source code. The annual PWM mass concentrations of each observed compound are additionally provided in Supplementary Figure 5b (gridded population from the Gridded Population of the World Database[1]). These indicate that the smallest model NMB's are found for the compounds that contribute to the largest fraction of total $PM_{2.5}$ mass. As further shown in the right panel of Supplementary Figure 5b, there is general agreement in the fractional contributions of each chemical compound to total $PM_{2.5}$ mass, providing confidence in the model's ability to accurately predict changes in the chemical production of $PM_{2.5}$ under various emission sensitivity simulations.

Lastly, total $PM_{2.5}$ mass concentrations are calculated using modeled output mass concentrations of aerosol nitrate (NO_3^-), sulfate (SO_4^{2-}), ammonium (NH_4^+), sea salt, dust, organic mass, and black carbon, as described in the analysis scripts package (https://github.com/emcduffie/GBD-MAPS-Global). In this work, spatially gridded annual total $PM_{2.5}$ mass concentrations are calculated by averaging monthly $PM_{2.5}$ concentrations. National-level $PM_{2.5}$ concentrations are averaged over the grid cells within each country's geographical borders.

Supplementary Text 4. Observational $PM_{2.5}$ Dataset for Model Evaluation

Evaluation of base model performance is a vital component of any analyses that derives results from modeled emission sensitivity simulations. In this work, the base 2017 GEOS-Chem $PM_{2.5}$ simulation and downscaled $PM_{2.5}$ exposure estimates (Fig. 1) are evaluated against a dataset of annual-average surface observations of total $PM_{2.5}$ mass and $PM_{2.5}$ chemical composition (where available). This section describes methods used to develop this observational dataset.

Supplementary Figure 4 provides a map of the individual measurement sites in this dataset, colored by their measurement networks. More extensive sampling and analysis details are reported from the North American networks than those from other regions, which have larger uncertainties due to a lack of consistent reporting on sampling and analysis protocols (particularly for EMEP and WHO datasets). Based on available reported metadata, some networks also provide mass concentrations derived from multiple sampling and analysis methods. Here we attempt to reduce sampling differences by selecting for consistent analysis methods between sites and networks when this information is available (described in detail below). Observational data also include uncertainties in the amount of aerosol water assumed in the gravimetric analysis of $PM_{2.5}$ and degree of volatilization of $PM_{2.5}$ and its chemical components (particularly ammonium nitrate and organics) during sampling and/or filter transport. Uncertainties in these observational datasets should be considered when comparing to modeled results.

2017 $PM_{2.5}$ Surface Observation Network Sites

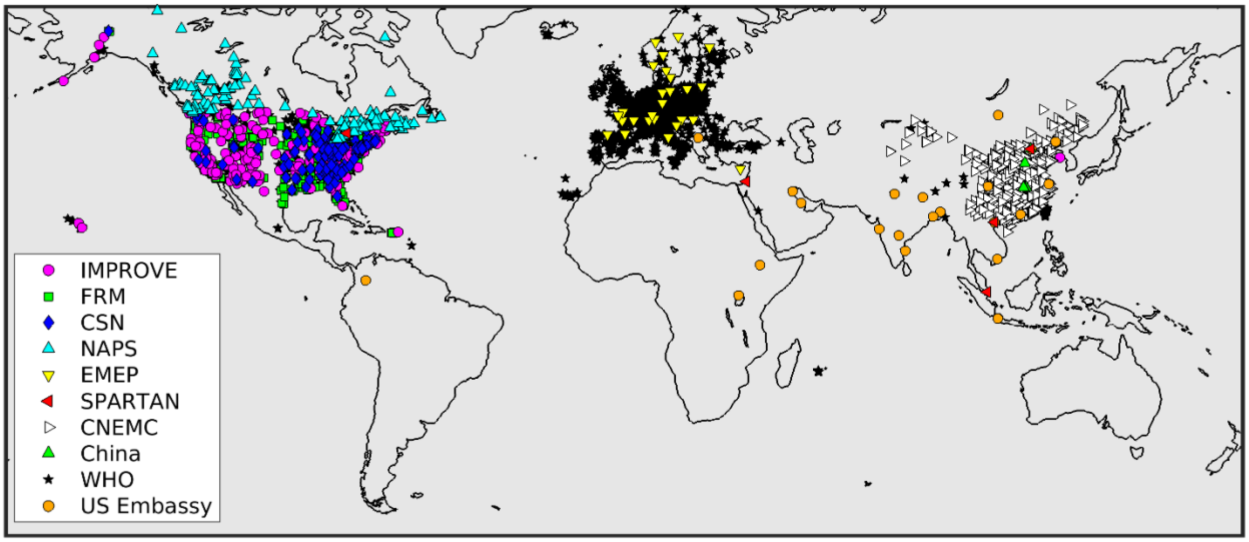

Supplementary Figure 4: Map of 2017 long-term $PM_{2.5}$ sampling stations. Symbol colors and shapes reflect individual monitoring networks. This figure includes sites that are used both for the total $PM_{2.5}$ mass and speciated $PM_{2.5}$ mass model evaluations.

The following sub-sections describe the individual networks that provide long-term measurements of $PM_{2.5}$ total mass and/or $PM_{2.5}$ chemical components. Estimates of $PM_{2.5}$ chemical composition in 2017 are available for a more select number of sites than total $PM_{2.5}$ mass, as shown in Supplementary Figure 5a. These sites are primarily in populated regions throughout North America, Europe, select sites in China, and 5 international sites through the SPARTAN network[34,35]. Data from the SPARTAN network and the compiled speciated inventory for China are used for the speciated comparison in Supplementary Figure 5b and are not used in Fig. 1. Annual average values from each site are calculated from the calculated monthly averages. Dataset development details can be found in the analysis scripts package at: https://github.com/emcduffie/GBD-MAPS-Global.

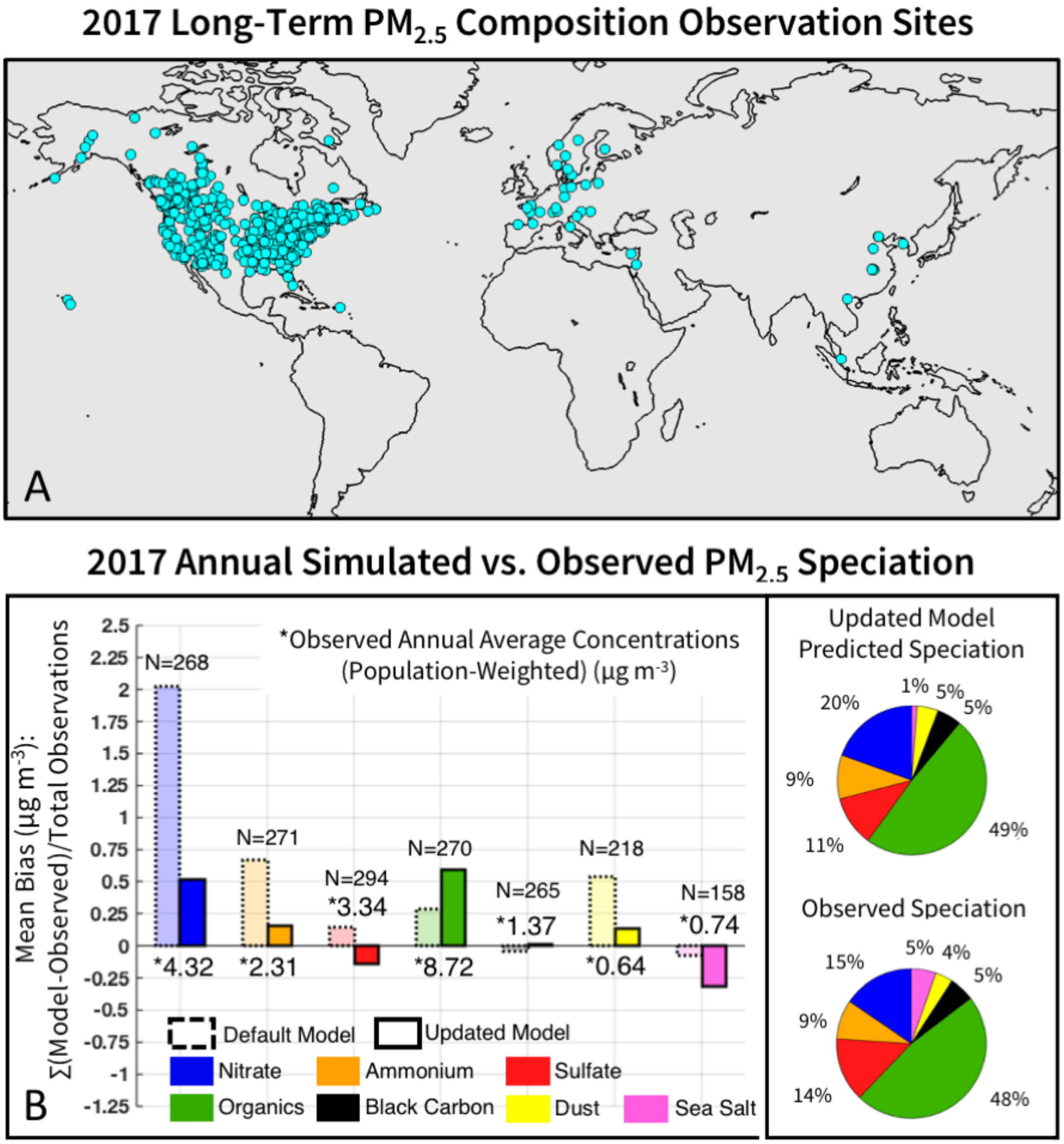

Supplementary Figure 5: Evaluation of default and updated base model simulation of $PM_{2.5}$ chemical components. (A) map of available long-term surface observations of $PM_{2.5}$ chemical components. (B) Bar plot of the normalized mean bias between the simulated and observed concentrations of individual $PM_{2.5}$ components (nitrate, ammonium, sulfate, organic aerosol, black carbon, dust, and sea salt). Light bars represent the values from the default v12.1.0 GEOS-Chem model. Darker bars show results from the updated model source code used here. Population-weighted average observed concentrations of each component are also provided. Pie charts illustrate the fractional distribution of $PM_{2.5}$ components from the updated model (top) and the observations (bottom).

WHO (World Health Organization) Compilation – Global

The World Health Organization has compiled an extensive database of annual-average surface-level $PM_{2.5}$ concentrations from around the world. In this work, these data are downloaded from https://www.who.int/airpollution/data/cities/en/. Only direct $PM_{2.5}$ measurements are used here, not those calculated from PM_{10} measurements (as in the GBD exposure calibration procedure), due to uncertainties in this conversion. This dataset is filtered to only include annual average measurements from 2017 for sites that report at least 75% measurement coverage. Data sources are available in the downloaded dataset, but measurement methods and analysis techniques are not readily available for the reported observations. We assume that data are reported at 35% RH and at local temperature and pressure. Observations from other regional networks may also be included in this compiled dataset, especially over North America and Europe. To ensure that these sites are not double counted in the model evaluation, we remove sites from the WHO dataset that are within 0.1° of other network sites.

US Embassy Measurements – Global

The U.S. Department of State collects air quality monitoring data from U.S. embassies and consulates around the world and has partnered with the U.S. Environmental Protection Agency to report data at AirNow.gov. Hourly observations from Beta-Attenuation Monitors (BAMs) are available for 28 sites in 2017 (55 sites by 2020). Hourly data for each site are downloaded from: https://www.airnow.gov/international/us-embassies-and-consulates. In this work, hourly raw concentrations are averaged into annual values and filtered to remove sites with <75% temporal data coverage. We assume that data are reported for local conditions (ambient pressure and temperature) and 35% RH.

CNEMC (China National Environmental Mentoring Centre) – China

The government of China has facilitated the deployment of nearly 2000 sites that measure $PM_{2.5}$ mass and its chemical composition. At the time of this study, only total $PM_{2.5}$ mass concentrations were publicly available. Data can be downloaded from: http://www.cnemc.cn/en/ and are available from May 2014 onward. Both Thermo Fisher Tapered Element Oscillating Microbalance (TEOM) 1405F analyzers and BAMs are used for continuous sampling of $PM_{2.5}$ mass, reported at each site at hourly time resolution. As

described in Wu, et al. [36], both methods use heaters to reduce the humidity in sampled air. This heating, however, can lead to mass loss due to the volatilization of $PM_{2.5}$ components. As a result, previous studies have reported lower total $PM_{2.5}$ mass measurements from TEOM instruments relative to US Federal Reference Methods (FRM)[37], largely as a result of the loss of semi-volatile compounds[38] and particularly in cold ambient temperatures[39]. To minimize this potential under-reporting, the 1405F monitoring system additionally measures concentrations in the volatilized portion of air, while a smart heater is used with the BAMs to minimize heating while also controlling the RH of the sample at 35%[36]. In this work, we assume that all CNEMC mass concentrations are reported at 35% RH and at local temperature and pressure, consistent with other networks. Monthly averages for January-December 2017 are then calculated for each site that reports a complete number of 24 measurements each day, for at least 20 days each month (~75% temporal coverage each month).

FRM Sites (Federal Reference Method) – United States

FRM sites follow protocols specified in Appendix L to Part 50 of Title 40 in the United States Code of Federal Regulations (CFR) – *Reference Method for the Detection of Fine Particulate Matter as $PM_{2.5}$ in the Atmosphere*. These sites measure total $PM_{2.5}$ mass by gravimetric analysis of a Teflon collection filter. Samples are collected on a filter for a 24-hour period every 3rd day, then transported to an analysis facility where filters are allowed to equilibrate for a minimum of 24 hours prior to weighing. The temperature and RH must be controlled between 20-23°C and 30-40%, respectively during the analysis. All data are reported at local ambient conditions (pressure and temperature). For this work, FRM data were downloaded from: http://views.cira.colostate.edu/fed/QueryWizard/, with additional details in the analysis scripts package. Data are saved as monthly averages for all sites with at least 10 measurements during the month (8 for February).

IMPROVE (Interagency Monitoring of Protected Visual Environments) – United States

Details about the IMPROVE network are reported elsewhere[40] and at http://vista.cira.colostate.edu/Improve/. IMPROVE sites are generally focused on rural areas and follow the same sampling procedures at each site. Briefly, each site has four measurement modules:

1) Teflon filter for gravimetric mass and X-ray fluorescence (XRF) analysis of trace elements

2) denuder + Nylon filter for anions by ion chromatography

3) Quartz filter for organic carbon by thermal optical reflectance (TOR) and calculation of elemental carbon using HIPS (Hybrid Integrating Plate and Sphere system).

4) Coarse mode sampler

Samples are collected for 24 hours, every 3rd day, after which they are transported to laboratories for analysis (without controlling for temperature or pressure). Samples are allowed to equilibrate for a few minutes prior to sampling. Data are reported at local, ambient conditions (pressure and temperature). For this work, IMPROVE data were downloaded from: http://views.cira.colostate.edu/fed/QueryWizard/, with additional file formatting details listed in the analysis scripts package. Data for each compound are saved as monthly averages for all sites with at least 10 daily measurements during the month (8 for February). In this work, spatially and seasonally varying OM:OC ratios are used to convert total monthly organic carbon measurements to total organic mass. Ammonium is re-constructed from sulfate and nitrate ion measurements, assuming pure ammonium nitrate and ammonium sulfate in the aerosol phase. Dust is reconstructed from trace elements assuming normal oxides in typically occurring soil dust following Supplementary Eq. 1. Sea salt is calculated as 1.8*chloride following White [41]. Black carbon is taken as elemental carbon.

$$\text{Dust} = 2.2 \times \text{Al} + 2.49 \times \text{Si} + 1.63 \times \text{Ca} + 2.42 \times \text{Fe} + 1.94 \times \text{Ti} \qquad \text{(S-Eq. 1)}$$

CSN (Chemical Speciation Network) – United States

Details about the CSN network are reported elsewhere[42]. In contrast to IMPROVE, CSN sites focus primarily on urban areas and do not use the same sampling instrumentation/methods at each site. In general, collection and analysis methods are similar to those listed for the IMPROVE network, with the analysis of some species (i.e., organic and elemental carbon) following IMPROVE protocols. Similar to IMPROVE, samples are collected for 24 hours, every 3rd day. Samples are transported overnight, held at a temperature of $\leq 4°C$ to minimize sample volatilization. Gravimetric analysis of $PM_{2.5}$ follows the FRM protocol where the samples are allowed to equilibrate for 24 hours prior to analysis and measured in a controlled clean room between 20-23°C and 30-40% RH. All data are reported at local, ambient conditions (pressure and temperature). For this work, CSN data were downloaded from: http://views.cira.colostate.edu/fed/QueryWizard/, with additional details in the analysis scripts package. Data for each compound are saved as monthly averages for all sites with at least 10 daily measurements during the month (8 for February). The final calculation of

organic mass, black carbon, sea salt, and dust follow the procedures used for IMPROVE data, described above.

NAPS (National Air Pollution Surveillance Program) – Canada

The NAPS network was designed to provide long-term air quality data across populated regions in Canada. The NAPS network includes sites with 24-hour integrated measurements of $PM_{2.5}$ mass and its components every 3-6 days, as well as sites with continuous, hourly $PM_{2.5}$ measurements. Data are available at: http://data.ec.gc.ca/data/air/monitor/national-air-pollution-surveillance-naps-program/Data-Donnees/2017/?lang=en. In 2017, hourly measurements were reported from a variety of instruments including the TEOM, Scientific Synchronized Hybrid Ambient Real-time Particulate (SHARP) model 5030, and Met-One BAM. Additional integrated $PM_{2.5}$ zip files contain Excel files for each monitoring site across Canada. Integrated daily filters are collected for 24 hours every 3-6 days and are allowed to equilibrate in the laboratory prior to sampling. The temperature and humidity are controlled during weighing between 20-26°C and 37-47% RH, respectively. Some sites have a dual Teflon-nylon filter collection system to collect nitrate loss during sampling. Due to known losses of ammonium nitrate on Teflon filters, only nitrate data from sites with a dual filter cartridge are used in this analysis. For these sites, total nitrate is calculated as the sum of nitrate measured by IC from the Teflon filter and nitrate and nitrite collected from the Nylon filter and analyzed by IC. Data for each compound are saved as monthly averages for all sites with at least 5 daily measurements during the month (4 for February). For continuous $PM_{2.5}$ data, hourly data are also averaged for months with at least 5 sampling days and 24 hours of valid measurements each day. All data are reported at local, ambient conditions (pressure and temperature). In this work, concentrations of organic mass, dust, sea salt, nitrate, ammonium, and sulfate are calculated as for the IMPROVE and CSN data, described above. Black carbon is calculated from the difference between total carbon and organic carbon.

EMEP (European Evaluation and Monitoring Program) – Europe

Measurements collected by partner organizations across Europe are reported to the EMEP database. Despite standard metadata protocols, lack of consistent compliance has resulted in largely unknown sampling methods and analysis protocols for the data available from this network. For this work, the EMEP dataset was downloaded from http://ebas.nilu.no, with

additional details in the analysis script package. Due to limited measurements of silicon, dust is not calculated from this dataset. Reported sampling frequencies range from 1 hour to 1-month. Where metadata is available, measurements have been filtered for data greater than reported detection limits. Due to a lack of consistent reporting of meta-data, there are a large number of uncertainties in the data from this network including measurement methods, sample filter types, or whether data have been corrected to standard or local conditions. Here, we assume data are reported in local conditions for consistency in units with national ambient air quality standards and do not apply any other filters. Data for each compound are then standardized to a time series of daily averages and saved as monthly averages for all sites with at least 4 daily measurements during the month [43]. In this work, concentrations of organic mass, sea salt, nitrate, ammonium, and sulfate are calculated as for the IMPROVE, CSN, and NAPS data, described above. Black carbon is taken as elemental carbon.

Compiled $PM_{2.5}$ Mass and Chemical Components - China

Additional total $PM_{2.5}$ mass and chemical composition data for select sites in 2017 in China have been compiled from literature sources. The data used in this work include measurements from [44-48], which report data from 14 measurement locations throughout Beijing, Hebei, Zhejiang, Jiangsu, Shaanxi, and Inner Mongolia provinces, collected at various times during the period between August 2016 and February 2018. Measurement techniques for OC and BC include DRI- Thermal/optical carbon analyzers, while sulfate, nitrate, and ammonium measurement methods include IC. Additional sampling and analysis details for each study are provided in the above references. Available reported data were compiled into annual averages for each measurement site for comparison with model results.

SPARTAN (Surface Particulate Matter Network) – Global

An overview of the SPARTAN network is available in Snider, et al. [34], Weagle, et al. [35], and McNeill, et al. [49]. The SPARTAN network provides publicly available data for $PM_{2.5}$ total mass, chemical composition, and optical characteristics in populated regions of the world where air quality monitoring has been historically limited. SPARTAN reported complete yearly data from 5 sites in 2017 and has since grown to report data from 21 sites worldwide. SPARTAN monitors sample ambient air on a Teflon filter, intermittently for a total of 24 hours over a 9-day period, following protocols described in Snider, et al. [34]. Filters are then shipped in sealed containers at ambient temperature to analysis labs in North America where

they undergo analysis for total gravimetric $PM_{2.5}$ mass and concentrations of ions, equivalent black carbon, and trace elements. Particle bound water is calculated and residual mass (i.e., total $PM_{2.5}$ – inorganic mass + particle bound water) is considered to be organic mass. Total $PM_{2.5}$ mass is measured gravimetrically following EPA protocols under laboratory conditions where temperature and RH are controlled to between 20-23°C and 30-40%, respectively. Equivalent black carbon is measured by light absorbance with a smoke stain reflectometer, calibrated to co-located TOR measurements on a quartz filter. More recent analysis techniques for SPARTAN filters include XRF, HIPS, UV-Vis[49,50], the measurement of organics through FT-IR[51], and ongoing research activities to measure organic spectra through Aerosol Mass Spectroscopy[52]. For this work, SPARTAN data have been downloaded from: https://www.spartan- network.org/data, with additional details in the analysis scripts package. Due to known loss of ammonium and nitrate on Teflon filters, these compounds are not used from SPARTAN. Data for each compound are saved as monthly averages for all sites with at least two reported measurements. Dust is reconstructed as 10*([Al]+[Mg]+[Fe]) following Weagle, et al. [35]. Sea salt is calculated as 2.54*[Na]-0.1[Al] following Weagle, et al. [35]. Black carbon is taken as equivalent black carbon.

Supplementary Text 5. Model Input Emission Details

Global input emissions for the GEOS-Chem model are primarily for the year 2017 from the Community Emissions Data System[53], updated for the GBD-MAPS project: $CEDS_{GBD\text{-}MAPS}$[54,55]. The $CEDS_{GBD\text{-}MAPS}$ emissions dataset is available on Zenodo[56] and uses contemporary energy consumption data from the International Energy Agency, source and fuel-specific emission factors, as well as a mosaic scaling approach to incorporate global emission estimates (such as the EDGAR v4.3.2[57] inventory and ECLIPSEv5a inventory from the GAINS model[58,59]) with regional inventories to calculate monthly emission fluxes (kg m^{-2} s^{-1}) of key atmospheric pollutants (NO_x, SO_2, CO, speciated NMVOCs, NH_3, BC, and OC). Emissions are disaggregated into contributions from 11 anthropogenic source sectors and 4 fuel categories (solid biofuel, total coal, the sum of liquid fuel and natural gas, and all other non-combustion sources). CEDS emissions are gridded at a 0.5°×0.5° spatial resolution and do not include vertical distribution information. Further details about this dataset are described in McDuffie, et al. [54] and Hoesly, et al. [53]. $CEDS_{GBD\text{-}MAPS}$ emissions for 2017 are incorporated into the GEOS-Chem model using the

HEMCO emissions module[60] and are systematically removed in sensitivity simulations by zeroing out individual sources. Monthly $CEDS_{GBD\text{-}MAPS}$ emissions are additionally distributed over a 24-hr period using sector-specific diel scaling factors, calculated from the U.S. NEI 2011v1 dataset, implemented in HEMCO by Travis, et al. [61].

Supplementary Table 2 provides details about the CEDS and non-CEDS emission sources used for model emission sensitivity simulations in the main text. Non-CEDS emission sources include dust emissions from windblown, fugitive, combustion, and industrial sources (AFCID), as well as emissions from aircraft, open fires, volcanoes, lightning, the ocean, and biogenic sources. If emissions from 2017 are not available from a given source, the latest available year is used. Supplementary Figure 6 shows the total global annual emissions of NO_x, SO_2, CO, NH_3, OC, BC, total NMVOCs, and fine dust used in the base 2017 GEOS-Chem simulation. The categories shown in Supplementary Figure 6 correspond to the emission sensitivity simulation categories in Supplementary Table 2.

We also note that the fuel-specific contributions in this work may be lower estimates as some sub-sectoral emission categories were not assigned to a particular combustion fuel-type in the emissions dataset, as shown in Table 2 in McDuffie, et al. [54]. For example, contributions from fuel production, flaring, transformation, and fossil-fuel fires in the energy sector were not assigned to a combustion fuel-type. As a result, $PM_{2.5}$ contributions from these sources were included in the 'other sources' fuel category in Figs. 2-5 rather than the O&NG category. These contributions, however, were included in the total energy sector fractional results in these same figures. In addition, the emissions dataset does not include primary emissions of $PM_{2.5}$ associated with road, tire, and brake wear or ash from coal combustion. While the former source has been shown to have relatively small global $PM_{2.5}$ contributions [59], both sources may be important in regions with large fractional contributions from transportation and coal use.

Supplementary Table 2. Model emission sensitivity simulation descriptions. Includes emissions dataset references and descriptions. Note: "calculated" emissions depend on meteorological variables and are computed at the time of simulation.

#	Sector Sensitivity Simulation	Dataset	Year	Reference	Notes
1	**Agriculture (AGR)**<br>includes manure management, soil fertilizer emissions, rice cultivation, enteric fermentation, and other agriculture	$CEDS_{GBD-MAPS}$	2017	[54,56]	-
2	**Energy Production (ENE)**<br>Includes electricity and heat production, fuel production and transformation, oil and gas fugitive/flaring, and fossil fuel fires	$CEDS_{GBD-MAPS}$	2017	[54,56]	-
3	**Industry (IND)**<br>Includes Industrial combustion (iron and steel, non-ferrous metals, chemicals, pulp and paper, food and tobacco, non-metallic minerals, construction, transportation equipment, machinery, mining and quarrying, wood products, textile and leather, and other industry combustion) and non-combustion industrial processes and product use (cement production, lime production, other minerals, chemical industry, metal production, food, beverage, wood, pulp, and paper, and other non-combustion industrial emissions)	$CEDS_{GBD-MAPS}$	2017	[54,56]	-
4	**Road Transportation (ROAD)**<br>includes cars, motorcycles, heavy and light duty trucks and buses	$CEDS_{GBD-MAPS}$	2017	[54,56]	-
5	**Non-Road/Off-Road Transportation (NRTR)**<br>Includes Rail, Domestic navigation, Other transportation	$CEDS_{GBD-MAPS}$	2017	[54,56]	-
6	**Residential Combustion (RCO-R)**<br>includes residential heating and cooking	$CEDS_{GBD-MAPS}$	2017	[54,56]	-
7	**Commercial Combustion (RCO-C)**<br>Includes commercial and institutional combustion	$CEDS_{GBD-MAPS}$	2017	[54,56]	
8	**Other Combustion (RCO-O)**<br>Includes combustion from agriculture, forestry, and fishing	$CEDS_{GBD-MAPS}$	2017	[54,56]	-
9	**Solvents (SLV)**<br>Includes solvents production and application (degreasing and cleaning, paint application, chemical products manufacturing and processing, and other product use)	$CEDS_{GBD-MAPS}$	2017	[54,56]	-
10	**Waste (WST)**<br>Includes solid waste disposal, waste incineration, waste-water handling, and other waste handling	$CEDS_{GBD-MAPS}$	2017	[54,56]	-
11	**International Shipping (SHP)**<br>Includes international shipping and tanker loading	$CEDS_{GBD-MAPS}$	2017	[54,56,62,63]	**A**
12	**Agricultural Waste Burning (AGBURN)**<br>Includes open fires from agricultural waste burning	GFED4.1s	2017	[64,65]	B
13	**Other Open Fires (OBURN)**<br>Includes deforestation, boreal forest, peat, savannah, and temperate forest fires	GFED4.1s	2017	[64,65]	B
14	**Fugitive, Combustion, Industrial dust (AFCID)**	AFCID	2012, 2013, 2015	[66]	C
15	**Windblown Dust (WDUST)**	DEAD model	calculated	[67,68]	D
16	**Remaining Emission Sources (OTHER)**<br>Includes all remaining emission sources:				
	volcanic SO_2	AeroCom	2017		E
	aircraft	AEIC	2005	[69]	
	lightning NO_x	LightNOx	calculated	[70]	F
	biogenic Soil NO	Soil NO_x	calculated	[71]	G
	ocean	SeaFlux, GEIA, SeaSalt, Inorg_Iodine	calculated	[12,72-77]	H
	biogenic emissions	MEGANv2.1	calculated	[78]	I
	very short-lived iodine and bromine species	LIANG_ BROMOCARB ORDONEZ_ IODOCARB	2000	[79,80]	
	decaying plants	DECAYING_ PLANTS		[73]	
	**Fuel Sensitivity Simulations**	**Dataset**	**Year**	**Reference**	**Notes**
17	**Total Coal**<br>Includes hard coal, brown coal, coal coke	$CEDS_{GBD-MAPS}$	2017	[54,56]	-
18	**Solid Biofuel**	$CEDS_{GBD-MAPS}$	2017	[54,56]	-

	Includes solid biofuel				
19	**Liquid Oil and Natural Gas** Includes light and heavy oil, diesel oil, and natural gas	$CEDS_{GBD-MAPS}$	2017	[54,56]	-
20	**Process** Includes non-combustion CEDS 'process' source emissions.	$CEDS_{GBD-MAPS}$	2017	[54,56]	-
	**Sector & Fuel Sensitivity Simulations**	**Dataset**	**Year**	**Reference**	**Notes**
21	**Total Coal from Energy Production** Includes hard coal, brown coal, coal coke; Includes electricity and heat production.	$CEDS_{GBD-MAPS}$	2017	[54,56]	J
22	**Total Coal from Industrial Processes** Includes hard coal, brown coal, coal coke; Includes Industrial combustion (iron and steel, non-ferrous metals, chemicals, pulp and paper, food and tobacco, non-metallic minerals, construction, transportation equipment, machinery, mining and quarrying, wood products, textile and leather, and other industry combustion)	$CEDS_{GBD-MAPS}$	2017	[54,56]	K
23	**Total Coal from Residential Combustion (RCO-R)** Includes hard coal, brown coal, coal coke; Includes residential heating and cooking	$CEDS_{GBD-MAPS}$	2017	[54,56]	-
24	**Solid Biofuel from Residential Combustion (RCO-R)** Includes solid biofuel; Includes residential heating and cooking	$CEDS_{GBD-MAPS}$	2017	[54,56]	-

[A]CEDS International shipping emissions run with the PARANOX ship plume module, which calculates co-emitted concentrations of O_3 and HNO_3 in aged shipping plumes.

[B]Official GFED4 emissions have been released through 2016. 2017 and 2018 emissions are available through a beta release (https://www.geo.vu.nl/~gwerf/GFED/GFED4/) that provides updates to estimated monthly emissions of dry matter (DM) and carbon (C) based on the relationship between MODIS active fire detections and the GFED4s inventory for the years 2013-2016. To distribute monthly emissions, these files also include daily variability based on active fire distributions and diurnal cycles based on climatological data following the approach of Mu, et al. [65]. Emission factors for individual species (kg or kg C/ kg DM) are from the original GFED4s release.

[C]The AFCID (Anthropogenic Fugitive, Combustion, and Industrial Dust) inventory is based on 2015 global monthly average primary particulate matter emissions from the ECLIPSEv5a inventory and regional monthly mean inventories for 2013 over India and 2012 over China.

[D]Global windblown mineral dust emissions are calculated for the year 2017 using the dust entrainment and deposition (DEAD) model.

[E]Emissions obtained from NASA/GMAO and include contributions from eruptive and degassing volcanic emissions in 2017, details are here: http://ftp.as.harvard.edu/gcgrid/data/ExtData/HEMCO/VOLCANO/v2019-08/README

[F]Lightning emissions of NO match OTD/LIS climatological observations of lightning flashes from May 1995 – December 2013, as described by Murray et al., 2012.

[G]Soil NO_x emissions are calculated as a function of surface vegetation type, temperature, precipitation history, and a canopy reduction factor, following the parameterization described in Hudman, et al. [71]. Note that fertilizer emissions are not included in this calculation as fertilized soil emissions are included in the $CEDS_{GBD-MAPS}$ inventory.

[H]Ocean-air exchange fluxes for DMS, acetone, acetaldehyde, and inorganic iodine are from [74], [72], [73], and [77], respectively. Ocean emissions of NH_3 from natural sources are set to 1990 levels from GEIA and NH_3 emissions from artic seabirds are from [76] and [75]. Sea salt emissions are calculated following [12].

[I]Biogenic emissions are calculated using the Model of Emissions of Gases and Aerosols from Nature (MEGAN) v2.1.

[J]Energy emissions from fuel production and transformation, oil and gas fugitive/flaring, and fossil fuel fire emissions are not assigned to specific fuel types

[K]Industry emissions from non-combustion industrial processes and product use (cement production, lime production, other minerals, chemical industry, metal production, food, beverage, wood, pulp, and paper, and other non-combustion industrial emissions) are not assigned to specific fuel types.

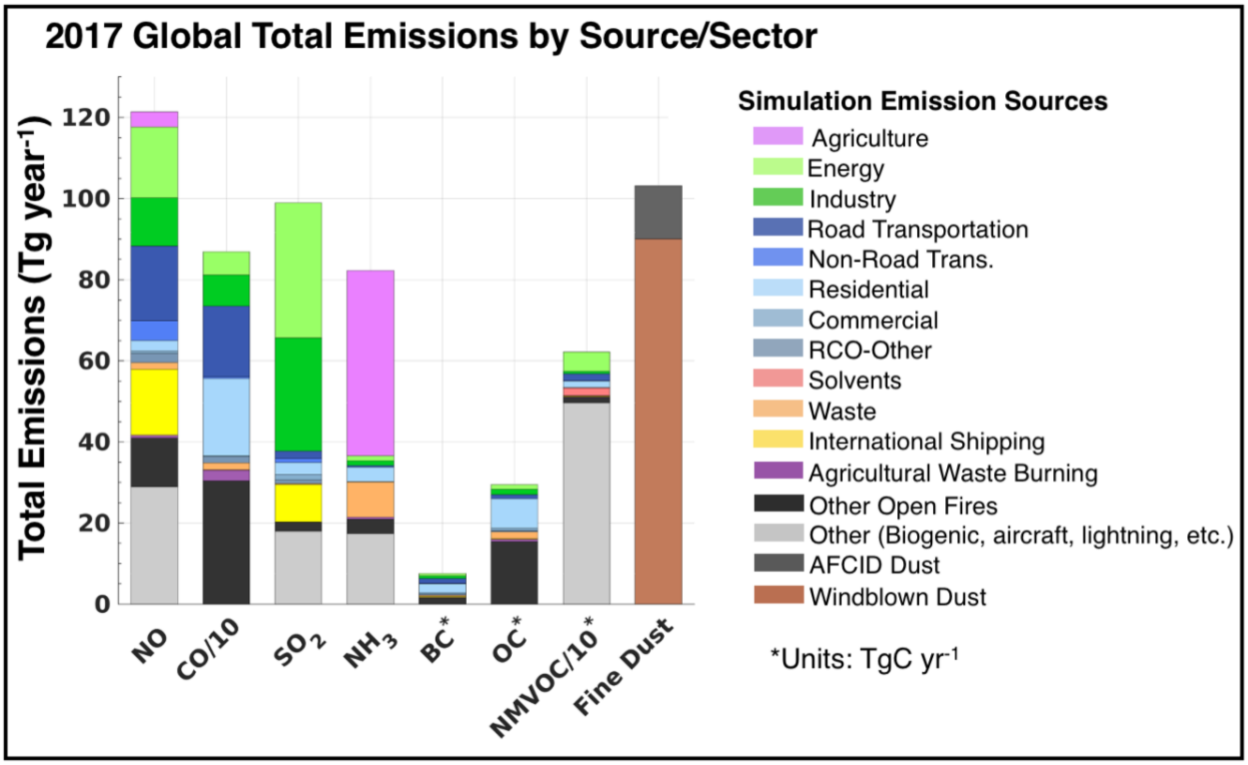

Supplementary Figure 6: Global total 2017 base simulation emissions. Emissions are colored by source sector category. BC, OC, and total NMVOCs are provided in units of Tg carbon yr^{-1}. Emissions of CO and NMVOCs are divided by 10 for illustration purposes only. Note: OC emissions do not include the GEOS-Chem chemical compound SOAP (secondary organic aerosol precursor), which is emitted from biogenic sources and co-emitted with CO. Emissions of this compound, however, are included in each model simulation.

Supplementary Text 6. Fractional Fuel and Sector Contributions – Additional Comparisons to Previous Nation-Level Studies

To provide further context for our results, this section provides further comparisons to previous national-level studies that have also used 3D chemical transport models to quantify $PM_{2.5}$ source contributions and/or the associated ambient $PM_{2.5}$ disease burden. Where available, we primarily compare the reported fractional contributions to minimize methodological and input data differences. As described in the main text, differences in sectoral definitions (e.g., including fires in the agricultural sector or waste in the residential sector) highlight the importance of clearly defining emission sector definitions in source contribution studies (e.g., Supplementary Table 2). This text is not an exhaustive review of previous studies.

Fuel Types

Previous studies using similar methodologies have typically combined all anthropogenic sources for global or regional scale analyses[81-83] or reported contributions from single or aggregate fuel-types[84], typically for select countries such as China and India[85-87]. Previous studies also provide estimates of years prior to 2017, which may not capture recent trends in $PM_{2.5}$ chemical precursor emissions, such as recent reductions in China[88].

Previous national-level studies have only investigated the contribution from coal combustion emissions for select countries and sectors. For example, previous studies have discussed the importance of residential, energy, and industrial coal use on local and regional $PM_{2.5}$ pollution in India[87] and China[86], particularly during winter[89,90]. Emission reduction policies in China have also recently targeted coal use in these sectors. Fractional coal contributions in the residential, energy, and industry sectors in this study in 2017 were estimated to account for 5.3%, 4.7%, and 9.1% of total $PM_{2.5}$ sources in China and 1.4%, 7.0%, and 8.2% in India. These are generally smaller than the corresponding contributions of 4%, 9%, and 17% in 2013 for China[86], but generally agree well with the 8% contributions from both energy and industrial coal use in India in 2015[87]. When considering total coal use across all sectors, these previous studies have estimated contributions of 40% in China and 16% in India[86,87]. In 2017, total coal contributions were lower in China (22.7%) and slightly larger in India (17.1%), which may be a result of different methodologies, but is also consistent with recent emission trends in these respective countries.

For solid biofuel, Chafe, et al. [84] previously investigated the contribution of residential cooking with biofuel to the $PM_{2.5}$ mass and associated burden at both global and regional scales for the year 2010. Both studies predict large relative contributions in South and Southeast Asia, though Chafe, et al. [84] also predict fractional contributions from biofuel cooking in Southern Sub-Saharan Africa and Southern Latin America that were more than twice as large as those in this work. Reductions in the proportion of the population using solid fuels in these locations between 2010 and 2017 may partially explain these differences. In China and India, two previous studies found that solid biofuel combustion for residential heating and cooking contributed to 15% in China in 2013[86] and 24% in India in 2015[87]. These fractional contributions were similar to those of 12.7% and 22.5% for China and India in 2017 and suggest that these relative contributions have been relatively constant in recent

years. Due to such persistent contributions, however, previous studies have also shown the potential for significant air quality benefits by addressing the fuels and combustion efficiencies of sources used for residential heating and cooking in China[89,90]. For 2017, we estimate a total of nearly 250,000 (95% CI: 189,500-305,500) deaths avoidable by eliminating both solid biofuel and coal use in the residential sector in China.

Sectors

For the residential sector, previous studies for India estimated $PM_{2.5}$ disease burden contributions between 27% and 50% in 2010 and 2015[87,91]. Contributions in 2017 were comparable but slightly lower at between 23%-35% when comparable sub-sectors (e.g., residential + waste) were considered. In China, previous residential estimates ranged from 25%-32% in 2010[91,92] and ~19%-22% in 2013[86,93], both consistent with 26% here in 2017 (Supplementary Data 1). Fig. 2 and Fig. 3 in the main text show that the fractional residential contributions were generally smaller in Canada and the U.S. than in Asia, also consistent with multiple previous studies[91,94-98]. Emissions estimates from the residential sector, however, are particularly uncertain in emission inventories compared to those from other large anthropogenic emission sources[54,99-101].

For the energy and industry sectors, 2017 contributions were 10.2% and 11.7%, respectively. These sectors have been studied relatively extensively in past work compared to other $PM_{2.5}$ sources, however, differences may arise due to differences in the detailed sub-sectoral categories used here, or recent emission changes. The more detailed sectors such as commercial, AFCID, and waste examined here isolate sources that may have either been lumped into the industry sector in prior work or neglected altogether. Over recent years, industrial and energy emissions have been decreasing in China, while these emission sources have been simultaneously increasing throughout other parts of Asia, Latin America, and Africa[54]. Previous studies have specifically investigated these source contributions at the national level in China[86,91-93], India[87], Canada[94], the U.S.[98], and throughout Africa[82,83]. In Canada, combined fractional energy and industry contributions in this work were similar to previous results[94]. In China, previous energy contributions were consistent with this work, however industrial emissions in 2017 were close to half those previously reported for 2010 and 2013[86,92,93]. In Africa, two recent studies found the greatest contributions from the energy sector in Egypt and Southern Sub-Saharan Africa[82,83], also consistent with this work (Fig. 3).

Two additional fuel and sector specific simulations for 2017 revealed that 91% of these energy contributions in South Africa were from coal, while only 46% were from coal in Egypt (Fig. 3, Supplementary Data 1). One additional study predicted a much smaller (3%) contribution from combined energy and industry sources in Egypt in 2010, predicting instead a 92% contribution from natural sources[91]. More detailed assessments of these differences between industry, energy, and other source contributions are largely limited by a lack of more detailed emission sector descriptions.

For dust, agriculture, transportation, and fires, agreement with previous national-level results were variable. For example, national-level fractional dust estimates in 2017 were much larger for North Africa, the Middle East[102], and China[93], and smaller in India[87] compared to previous studies. For India specifically, updates to the model deposition[29] and dust size distribution schemes[14], as well as interannual variability in dust emission fluxes and removal rates, likely contribute to the smaller total contribution from fine dust (< 2.5 μm diameter) in this work (~14.9%) relative to previous estimates (~38%), derived using an older version of the GEOS-Chem model. As shown in Supplementary Figure 5, the model updates in this work improved the agreement with surface dust observations, however, the measured PWM dust concentrations at surface monitors were < 1 $\mu g/m^3$, indicating that current surface monitor locations may not provide an accurate characterization of the total population exposure to dust. These uncertainties highlight the need for increased monitoring and continued improvement to the model treatment of dust to improve the accuracy of contribution estimates from this source.

For non-combustion agriculture, 2017 estimates were generally smaller than previous regional-level studies[103,104]. For example, 2017 contributions in Europe, North America, and South and East Asia were less than 22%, 11%, and 12%, respectively, while the same source was estimated to contribute to 34%, 17%, and 10% in 2010[103]. Global and regional NH_3 emissions have been increasing between 2010 and 2017[54], indicating that differences here are methodological (e.g., differences in emissions or chemical production), rather than real temporal trends. Our mechanistic updates to the ammonium nitrate simulation (Supplementary Text 3) both improved the agreement with observations and reduced the agricultural sources of $PM_{2.5}$ in this work.

For the transportation sector, relative contributions have also been previously investigated at the national scales[105-107]. Consistent with previous studies, total transportation contributions in this work were generally greater than the global average in North America, Europe, parts of Asia, Australia, and Latin America[105-107]. Comparisons with additional national-level results[86,87,91-93,98,107] show that differences in fractional contributions generally follow recent trends in transportation emissions, with recent decreases in China and the U.S. and increases in India.

For fire emissions, comparisons to previous global and national level estimates are discussed in the main text.

Waste, solvent use, and international shipping sectors have relatively small contributions on the global scale but can significantly contribute to national and sub-national $PM_{2.5}$ variation (e.g., up to 18% in Sri Lanka; Fig. 5). Contributions from waste combustion to the ambient $PM_{2.5}$ disease burden have not been previously reported. The relative contribution from the solvent sector has only been reported in one previous national-level study[86]. As a result of non-linear $PM_{2.5}$ mass production, solvent emission reductions can result in an increase in total $PM_{2.5}$ mass. This was shown previously for a study in China[86]. Solvent emission reductions in 2017, however, resulted in a total mass decrease in this same country. As the solvent sector primarily emits NMVOCs (Supplementary Figure 6), this variable sign response demonstrates that decreases in these emissions can increase the availability of atmospheric oxidants, leading to increases in inorganic aerosol mass in NO_x-limited/VOC-saturated chemical regimes[108]. Therefore, this negative response may be important to consider when developing air pollution reduction strategies in regions with large VOC/NO_x emission ratios. Solvent emissions, however, are also highly uncertain as NMVOCs may be underestimated in U.S. emissions inventories by a factor of 2-3[109]. Relative national contributions from international shipping are generally consistent with previous studies[110,111], where the largest relative contributions are predicted in coastal countries such as Ireland, Portugal, and the Bahamas (more than 12% each in 2017).

Supplementary Text 7. Uncertainty Sensitivity Study

We conduct an additional sensitivity test to account for potential uncertainties in the $PM_{2.5}$ disease burden associated with the age- and disease-specific baseline mortality data from the

2019 GBD. The 95% uncertainty ranges are calculated by applying lower and upper estimates of the baseline mortality data to the PAF in Equation 2, derived from the mean CRF. The resulting 95% confidence intervals for 21 world regions are shown in Supplementary Figure 7, compared to the 95% CIs derived from uncertainties in the mean CRFs (reported in the Main Text). As the upper and lower limits in the baseline and CRF datasets are both estimated from multiple draws of underlying distributions, propagating the uncertainties from these two input variables likely leads to an overestimate in the 95% CI for the total attributable disease burden. Supplementary Figure 7 shows that for most regions, the 95% CI associated with uncertainties in the CRFs encompass the 95% CIs associated with uncertainties in the baseline mortality estimates. Additional uncertainties in the $PM_{2.5}$ exposure estimates and modeled fractional source contributions are not considered here to due computational limitations.

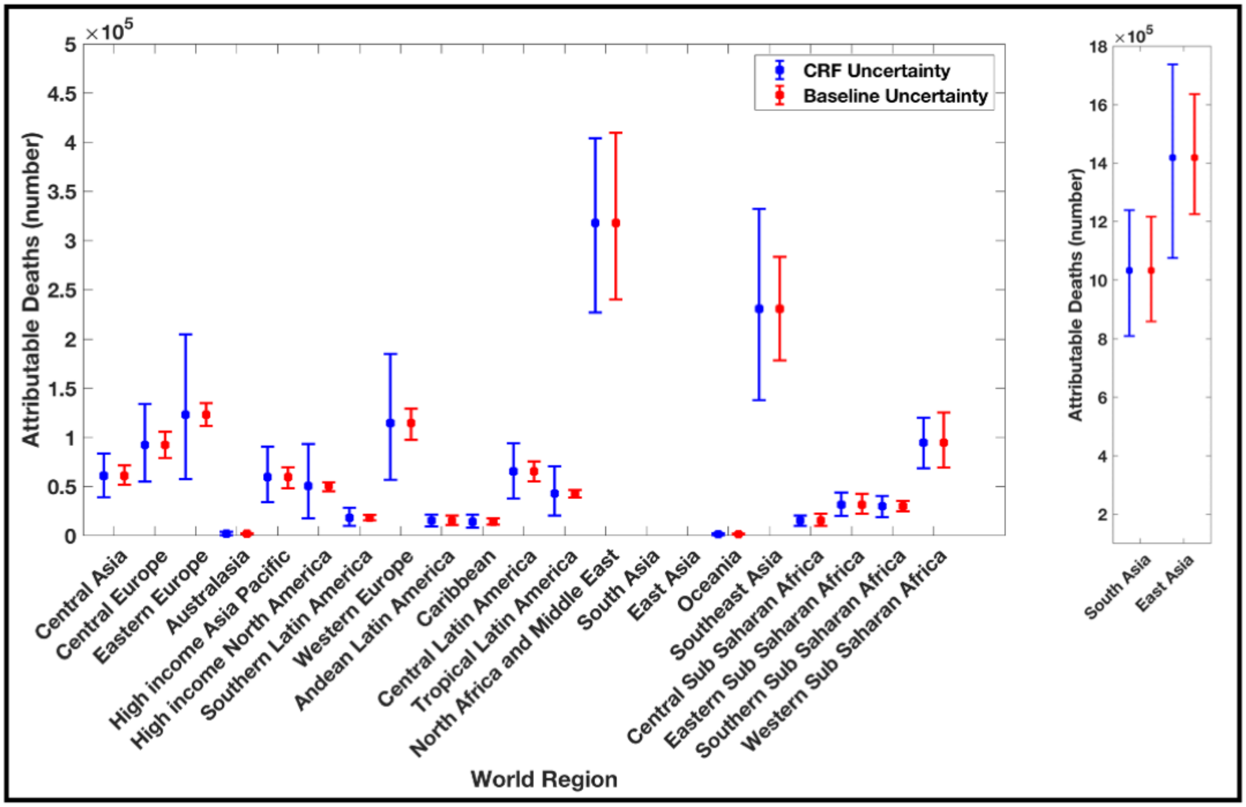

Supplementary Figure 7. Total disease burden estimates and confidence intervals for 21 world regions, derived from uncertainties in CRFs and baseline mortality data. Total disease burden estimates are from Supplementary Data 1. Uncertainty ranges illustrate the 95% CI derived from uncertainty estimates in the CRFs (blue) and baseline mortality data (red). The bounds for South and East Asia are shown on an expanded scale to the right.

Supplementary Text 8. Consideration of the Zero-Out (Brute Force) Method

Similar to many other analyses of this type of work, our analysis uses a zero-out or brute force approach for model sensitivity simulations. This widely used approach is designed to quantify source contributions to the total $PM_{2.5}$ disease burden under complete elimination of emissions from individual sources. As discussed in previous zeroing-out studies[35,91,92,103], the non-linear chemical production of $PM_{2.5}$ will result in the sum of individual $PM_{2.5}$ simulations to exceed the total $PM_{2.5}$ mass predicted in the base simulation. By implementing Eq. (5) in the Main Text, we ensure that fractional contributions from individual source sectors sum to 100% in this work. Using this approach, the final fractional $PM_{2.5}$ source contributions will be sensitive to the number of individual source sensitivity simulations that are included in the calculation, resulting in further differences between the detailed simulations in this work and previous similar studies. Due to this non-linearity, fractional and absolute contributions predicted from this method may not be consistent with simulations that implement more moderate reduction strategies (i.e., < 20-50% emission reductions), or strategies that simultaneously target multiple emission sectors (e.g., simultaneous reductions in both energy and industry sources).

Supplementary Text 9. Methodological Schematic

As described in the main text, results in this study are derived by integrating high-resolution satellite-derived $PM_{2.5}$ exposure estimates, CRFs from the 2019 GBD, and fractional source contribution results from 24 emission sensitivity simulations with the GEOS-Chem chemical transport model. Supplementary Figure 8 illustrates the overall workflow of this methodology. In Step 1, gridded global emissions of $PM_{2.5}$ precursors are developed as a function of source sector and fuel-type (Supplementary Table 5; anthropogenic emissions largely from the $CEDS_{GBD\text{-}MAPS}$ inventory), as described in McDuffie, et al. [54]. In Step 2, emissions are used as input in an updated version of the GEOS-Chem 3D chemical transport model (described in Supplementary Text 3), with the simulated $PM_{2.5}$ concentrations validated against available mass and composition surface observations (described in Supplementary Text 4). In Step 3, a series of zero-out emission sensitivity simulations are conducted (Supplementary Table 2) with the GEOS-Chem model and emission inputs. The resulting $PM_{2.5}$ concentrations from each simulation are compared to the base simulation (with all emission sources) to quantify the modeled fractional $PM_{2.5}$ contributions (reported in Date Files 1 (sectors) and 2 (fuel-types)). In

Step 4, high-resolution $PM_{2.5}$ exposure estimates are derived by downscaling exposure estimates from the 2019 GBD (described in Supplementary Text 10; reported in Supplementary Data 1 and 2) and are applied to the fractional model source contributions from Step 2 to quantify absolute source-specific contributions to ambient $PM_{2.5}$ mass. In Step 5, CRFs from the GBD2019 (Supplementary Figure 2) are combined with downscaled $PM_{2.5}$ exposure estimates from Step 4 to calculate the total ambient $PM_{2.5}$ disease burden (reported in Supplementary Data 1 and 2). The total burden is combined with modeled fractional source contributions from Step 3 to calculate source-specific burden contributions reported throughout the manuscript. Lastly, Step 6 highlights the data assets that are associated with this analysis and manuscript, including the analysis scripts, model source code, input emissions, CRFs, baseline burden and exposure estimate datasets (https://github.com/emcduffie/GBD-MAPS-Global), and the global, regional, national, and subnational source sector and fuel contribution results (Supplementary Data 1 and 2).

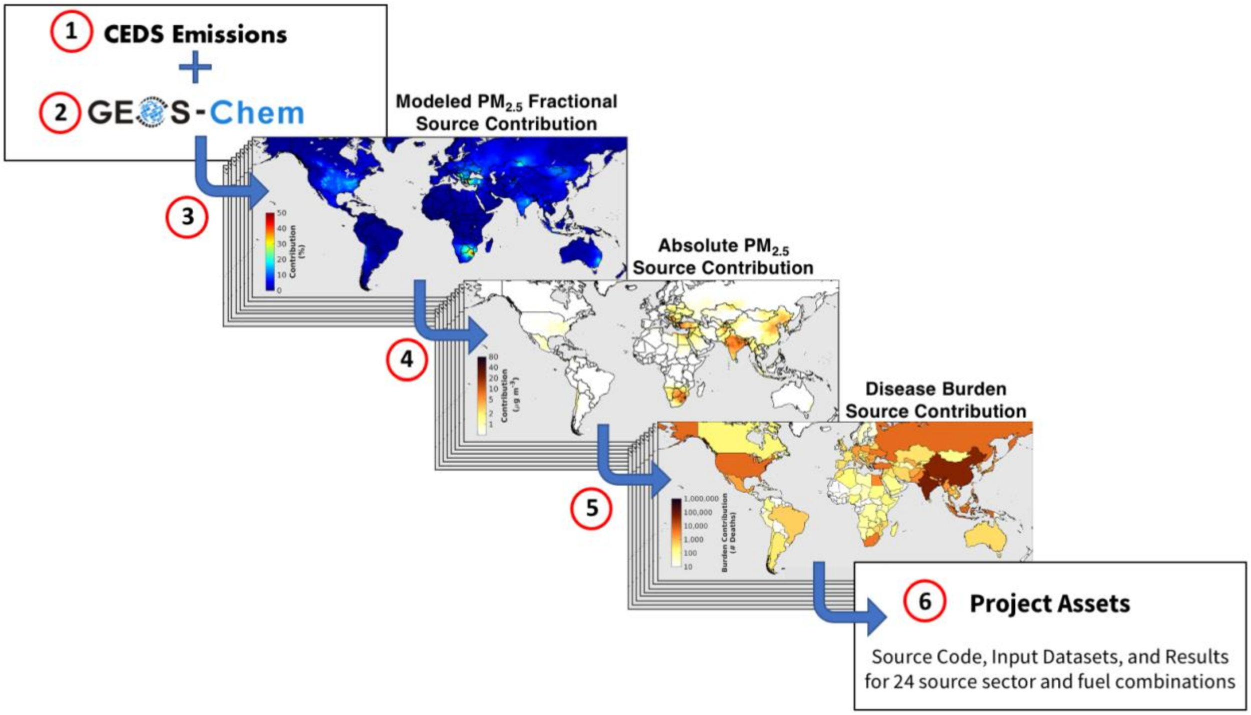

Supplementary Figure 8: Overall methodological workflow schematic. The relevant equations and data from Supplementary Text 9 are indicated in each step.

Supplementary Text 10. $PM_{2.5}$ Exposure Estimates - Spatial Downscaling Procedural Details

The process of spatially downscaling GBD $PM_{2.5}$ exposure estimates using a newly available high-resolution product from Hammer, et al. [5] involves three steps, illustrated in Supplementary Figure 9. First, the 0.1°×0.1° GBD product is re-gridded to a 0.01°×0.01° global grid, by setting each value in the new fine-resolution grid boxes (100 boxes) to the value from the corresponding coarser grid box in the original GBD product. Second, the fractional contribution of each grid-box in the 0.01°×0.01° Hammer, et al. [5], product is calculated relative to the average $PM_{2.5}$ across the surrounding 100 grid boxes. In the event that the Hammer, et al. [5] product does not report data for a particular grid box, the spatial fraction in that box is set to 1. Third, these resulting fractional contributions are multiplied by the GBD values from the 0.01°×0.01° $PM_{2.5}$ product from Step 1. This process is independent of the GEOS-Chem emission sensitivity simulations.

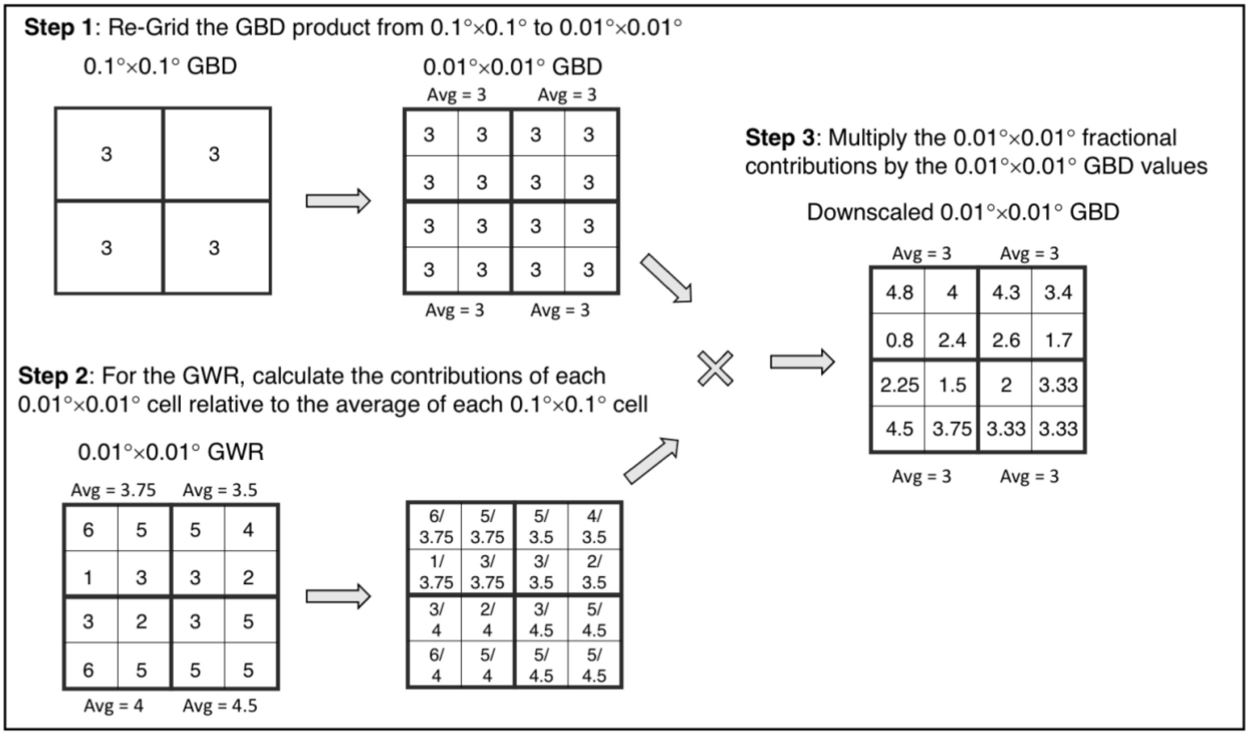

Supplementary Figure 9: Simplified schematic of the spatial downscaling procedure. Values in each example grid box represent example $PM_{2.5}$ mass concentrations in units of μg m^{-3}. In actuality, one of the 0.1°×0.1° grid boxes in Step 1 above corresponds to 100 grid boxes of

0.01°×0.01° resolution, not the four as shown here. In this figure 'GWR' refers to the high-resolution $PM_{2.5}$ estimates from Hammer, et al. [5].

To assess the sensitivity of $PM_{2.5}$ exposure estimates to the downscaling process, Supplementary Figure 10 shows maps of the gridded exposure estimates, as well as their correlations against observations for (A) the raw GEOS-Chem simulated concentrations, (B) original 0.1°×0.1° GBD $PM_{2.5}$ product, and (C) 0.01°×0.01° Hammer, et al. [5] product. Fig. 1 and Supplementary Figure 10 illustrate similarly good agreement between each of the exposure estimates and the total $PM_{2.5}$ mass observations, with correlation coefficients (r) and NMBs ranging from 0.89 to 0.979 and -3% to +11%, respectively. The added spatial information from the downscaling procedure slightly increases the NMB from +7% (Supplementary Figure 10b) to +11% (Fig. 1), but maintains a slightly higher correlation coefficient (r) than the high-resolution Hammer, et al. [5] product (0.977 vs 0.951). Across all four products, the 2017 global annual PWM $PM_{2.5}$ mass ranges between 31.3 $\mu g\ m^{-3}$ to 41.7 $\mu g\ m^{-3}$. Differences between the GBD and Hammer, et al. [5] products are largely due to different methods used to calibrate geophysical estimates to surface observations.

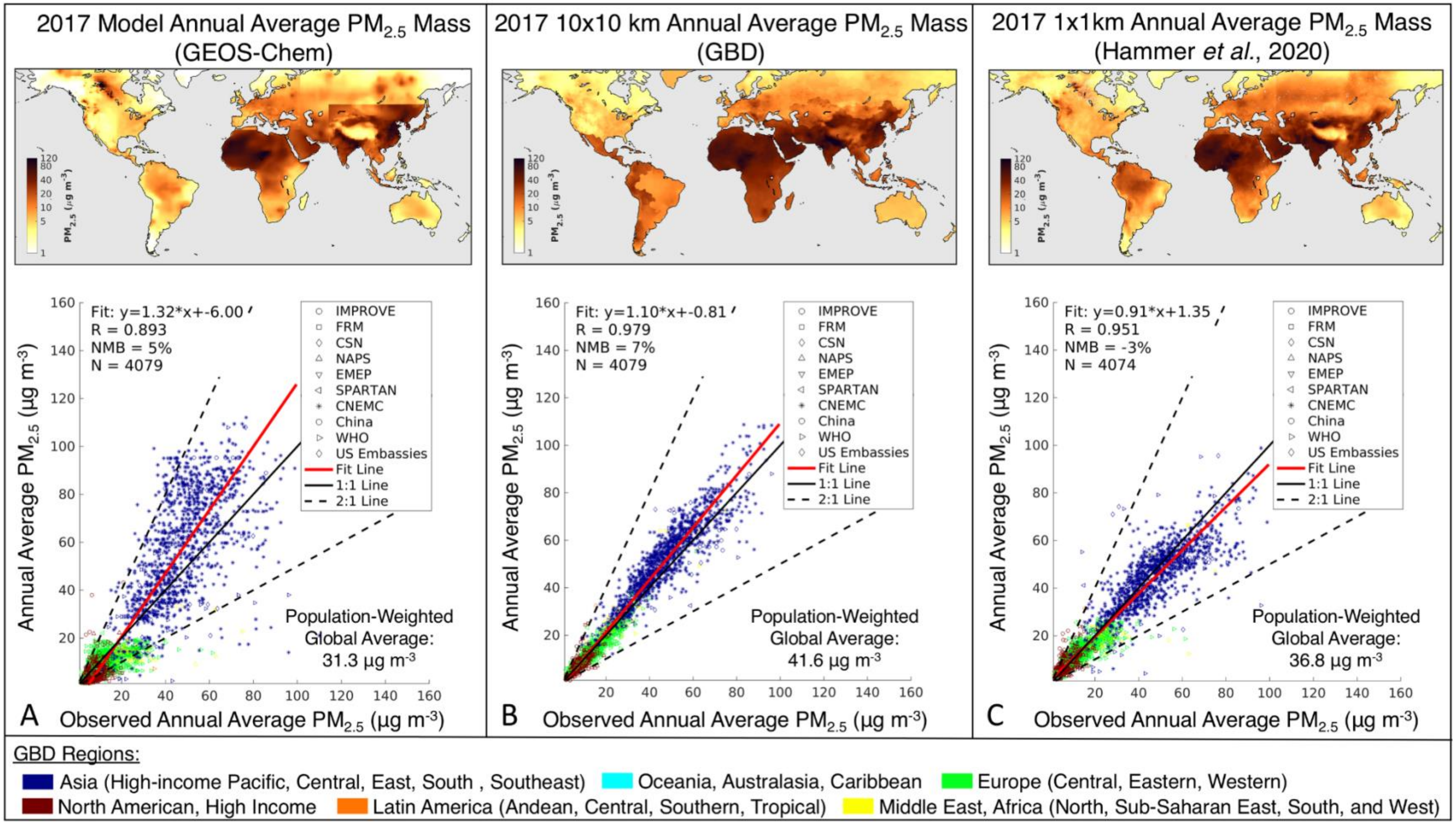

Supplementary Figuere 10: Comparison of three $PM_{2.5}$ exposure estimates against surface observations for the year 2017. (A) GEOS-Chem annual average $PM_{2.5}$ mass concentrations, (B) 0.1°×0.1° GBD annual average $PM_{2.5}$ exposure estimates, (C) 0.01°×0.01° Hammer, et al. [5] annual average exposure estimates. Each column contains a map of the $PM_{2.5}$ concentrations and a scatter plot comparing each product against 2017 surface observations. Colors represent world regions (Supplementary Table 1) and symbols represent observation networks (Supplementary Text 4). Red lines: correlation slope, solid black lines: 1:1 line, and dashed lines: 2:1 and 1:2 lines. The fit slope, intercept, correlation coefficient, normalized mean bias (NMB), number of observation points (N), and PWM $PM_{2.5}$ concentrations are also provided.

Supplementary References

1 CIESIN (Center for International Earth Science Information Network). Gridded Population of the World Version 4. (*Palisades, NY.*), doi:doi.org/10.1128/AAC.03728-14 (2017).

2 Burnett, R. *et al.* Global estimates of mortality associated with long-term exposure to outdoor fine particulate matter. *P. Natl. Acad. Sci.* **115**, 9592, doi:10.1073/pnas.1803222115 (2018).

3 Yin, P. *et al.* Long-term Fine Particulate Matter Exposure and Nonaccidental and Cause-specific Mortality in a Large National Cohort of Chinese Men. *Environmental Health Perspectives* **125**, 117002, doi:10.1289/EHP1673.

4 Hystad, P., Yusuf, S. & Brauer, M. Air pollution health impacts: the knowns and unknowns for reliable global burden calculations. *Cardiovascular Research*, doi:10.1093/cvr/cvaa092 (2020).

5 Hammer, M. S. *et al.* Global Estimates and Long-Term Trends of Fine Particulate Matter Concentrations (1998-2018). *Environ. Sci. Technol.*, doi:10.1021/acs.est.0c01764 (2020).

6 Bey, I. *et al.* Global modeling of tropospheric chemistry with assimilated meteorology: Model description and evaluation. *J. Geophys. Res. Atmos.* **106**, 23073-23095, doi:10.1029/2001JD000807 (2001).

7 Wang, Y. X., McElroy, M. B., Jacob, D. J. & Yantosca, R. M. A nested grid formulation for chemical transport over Asia: Applications to CO. *J. Geophys. Res. Atmos.* **109**, doi:10.1029/2004JD005237 (2004).

8 Philip, S., Martin, R. V. & Keller, C. A. Sensitivity of chemistry-transport model simulations to the duration of chemical and transport operators: a case study with GEOS-Chem v10-01. *Geosci. Model Dev.* **9**, 1683-1695, doi:10.5194/gmd-9-1683-2016 (2016).

9 Park, R. J., Jacob, D. J., Field, B. D., Yantosca, R. M. & Chin, M. Natural and transboundary pollution influences on sulfate-nitrate-ammonium aerosols in the United States: Implications for policy. *J. Geophys. Res. Atmos.* **109**, doi:10.1029/2003JD004473 (2004).

10 Wang, Q. *et al.* Global budget and radiative forcing of black carbon aerosol: Constraints from pole-to-pole (HIPPO) observations across the Pacific. *J. Geophys. Res. Atmos.* **119**, 195-206, doi:10.1002/2013JD020824 (2014).

11 Kim, P. S. *et al.* Sources, seasonality, and trends of southeast US aerosol: an integrated analysis of surface, aircraft, and satellite observations with the GEOS-Chem chemical transport model. *Atmos. Chem. Phys.* **15**, 10411-10433, doi:10.5194/acp-15-10411-2015 (2015).

12 Jaeglé, L., Quinn, P. K., Bates, T. S., Alexander, B. & Lin, J. T. Global distribution of sea salt aerosols: new constraints from in situ and remote sensing observations. *Atmos. Chem. Phys.* **11**, 3137-3157, doi:10.5194/acp-11-3137-2011 (2011).

13 Duncan Fairlie, T., Jacob, D. J. & Park, R. J. The impact of transpacific transport of mineral dust in the United States. *Atmos. Environ.* **41**, 1251-1266, doi:https://doi.org/10.1016/j.atmosenv.2006.09.048 (2007).

14 Zhang, L., Kok, J. F., Henze, D. K., Li, Q. & Zhao, C. Improving simulations of fine dust surface concentrations over the western United States by optimizing the particle size distribution. *Geophysical Research Letters* **40**, 3270-3275, doi:https://doi.org/10.1002/grl.50591 (2013).

15 Martin, R. V., Jacob, D. J., Yantosca, R. M., Chin, M. & Ginoux, P. Global and regional decreases in tropospheric oxidants from photochemical effects of aerosols. *J. Geophys. Res. Atmos.* **108**, doi:https://doi.org/10.1029/2002JD002622 (2003).

16 Koepke, P., Hess, M., Schult, I. & Shettle, E. P. Global Aerosol Dataset. (Max-Planck Institute for Meteorology, Hamburg, Germany, 1997).
17 Drury, E. *et al.* Synthesis of satellite (MODIS), aircraft (ICARTT), and surface (IMPROVE, EPA-AQS, AERONET) aerosol observations over eastern North America to improve MODIS aerosol retrievals and constrain surface aerosol concentrations and sources. *J. Geophys. Res. Atmos.* **115**, doi:https://doi.org/10.1029/2009JD012629 (2010).
18 Latimer, R. N. C. & Martin, R. V. Interpretation of measured aerosol mass scattering efficiency over North America using a chemical transport model. *Atmos. Chem. Phys.* **19**, 2635-2653, doi:10.5194/acp-19-2635-2019 (2019).
19 Lee, C. *et al.* Retrieval of vertical columns of sulfur dioxide from SCIAMACHY and OMI: Air mass factor algorithm development, validation, and error analysis. *J. Geophys. Res. Atmos.* **114**, doi:https://doi.org/10.1029/2009JD012123 (2009).
20 Ridley, D. A., Heald, C. L. & Ford, B. North African dust export and deposition: A satellite and model perspective. *J. Geophys. Res. Atmos.* **117**, doi:https://doi.org/10.1029/2011JD016794 (2012).
21 Hammer, M. S. *et al.* Interpreting the ultraviolet aerosol index observed with the OMI satellite instrument to understand absorption by organic aerosols: implications for atmospheric oxidation and direct radiative effects. *Atmos. Chem. Phys.* **16**, 2507-2523, doi:10.5194/acp-16-2507-2016 (2016).
22 Fountoukis, C. & Nenes, A. ISORROPIA II: a computationally efficient thermodynamic equilibrium model for K^+-Ca^{2+}-Mg^{2+}-NH_4^+-Na^+-SO_4^{2-}-NO_3^--Cl^--H_2O aerosols. *Atmos. Chem. Phys.* **7**, 4639-4659, doi:10.5194/acp-7-4639-2007 (2007).
23 Pai, S. J. *et al.* An evaluation of global organic aerosol schemes using airborne observations. *Atmos. Chem. Phys. Discuss.* **2019**, 1-39, doi:10.5194/acp-2019-331 (2019).
24 Lin, S.-J. & Rood, R. B. Multidimensional Flux-Form Semi-Lagrangian Transport Schemes. *Monthly Weather Review* **124**, 2046-2070, doi:10.1175/1520-0493(1996)124<2046:Mffslt>2.0.Co;2 (1996).
25 Wu, S. *et al.* Why are there large differences between models in global budgets of tropospheric ozone? *J. Geophys. Res. Atmos.* **112**, doi:10.1029/2006jd007801 (2007).
26 Lin, J.-T. & McElroy, M. B. Impacts of boundary layer mixing on pollutant vertical profiles in the lower troposphere: Implications to satellite remote sensing. *Atmos. Environ.* **44**, 1726-1739, doi:https://doi.org/10.1016/j.atmosenv.2010.02.009 (2010).
27 The International GEOS-Chem User Community. GEOS-Chem 12.1.0 (Version 12.1.0). Zenodo., doi:http://doi.org/10.5281/zenodo.1553349 (2018, November 26).
28 Heald, C. L. *et al.* Atmospheric ammonia and particulate inorganic nitrogen over the United States. *Atmos. Chem. Phys.* **12**, 10295-10312, doi:10.5194/acp-12-10295-2012 (2012).
29 Luo, G., Yu, F. & Schwab, J. Revised treatment of wet scavenging processes dramatically improves GEOS-Chem 12.0.0 simulations of nitric acid, nitrate, and ammonium over the United States. *Geosci. Model Dev. Discuss.* **2019**, 1-18, doi:10.5194/gmd-2019-58 (2019).
30 McDuffie, E. E. *et al.* Heterogeneous N_2O_5 Uptake During Winter: Aircraft Measurements During the 2015 WINTER Campaign and Critical Evaluation of Current Parameterizations. *J. Geophys. Res. Atmos.* **123**, 4345-4372, doi:10.1002/2018JD028336 (2018).
31 Shah, V. *et al.* Chemical feedbacks weaken the wintertime response of particulate sulfate and nitrate to emissions reductions over the eastern United States. *P. Natl. Acad. Sci.* **115**, 8110-8115 (2018).

32 McDuffie, E. E. *et al.* $ClNO_2$ Yields From Aircraft Measurements During the 2015 WINTER Campaign and Critical Evaluation of the Current Parameterization. *J. Geophys. Res. Atmos.* **123**, 12,994-913,015, doi:10.1029/2018JD029358 (2018).
33 Luo, G., Yu, F. & Moch, J. M. Further improvement of wet process treatments in GEOS-Chem v12.6.0: Impact on global distributions of aerosol precursors and aerosols. *Geosci. Model Dev. Discuss.* **2020**, 1-39, doi:10.5194/gmd-2020-11 (2020).
34 Snider, G. *et al.* SPARTAN: a global network to evaluate and enhance satellite-based estimates of ground-level particulate matter for global health applications. *Atmos. Meas. Tech.* **8**, 505-521, doi:10.5194/amt-8-505-2015 (2015).
35 Weagle, C. L. *et al.* Global Sources of Fine Particulate Matter: Interpretation of $PM_{2.5}$ Chemical Composition Observed by SPARTAN using a Global Chemical Transport Model. *Environ. Sci. Technol.* **52**, 11670-11681, doi:10.1021/acs.est.8b01658 (2018).
36 Wu, H. *et al.* Probabilistic Automatic Outlier Detection for Surface Air Quality Measurements from the China National Environmental Monitoring Network. *Advances in Atmospheric Sciences* **35**, 1522-1532, doi:10.1007/s00376-018-8067-9 (2018).
37 Sofowote, U., Su, Y., Bitzos, M. M. & Munoz, A. Improving the correlations of ambient tapered element oscillating microbalance PM2.5 data and SHARP 5030 Federal Equivalent Method in Ontario: A multiple linear regression analysis. *Journal of the Air & Waste Management Association* **64**, 104-114, doi:10.1080/10962247.2013.833145 (2014).
38 Tortajada-Genaro, L.-A. & Borrás, E. Temperature effect of tapered element oscillating microbalance (TEOM) system measuring semi-volatile organic particulate matter. *Journal of Environmental Monitoring* **13**, 1017-1026, doi:10.1039/C0EM00451K (2011).
39 Rizzo, M., Scheff, P. A. & Kaldy, W. Adjusting Tapered Element Oscillating Microbalance Data for Comparison with Federal Reference Method PM2.5 Measurements in Region 5. *Journal of the Air & Waste Management Association* **53**, 596-607, doi:10.1080/10473289.2003.10466196 (2003).
40 Hand, J. L., Prenni, A. J., Schichtel, B. A., Malm, W. C. & Chow, J. C. Trends in remote $PM_{2.5}$ residual mass across the United States: Implications for aerosol mass reconstruction in the IMPROVE network. *Atmos. Environ.* **203**, 141-152, doi:https://doi.org/10.1016/j.atmosenv.2019.01.049 (2019).
41 White, W. H. Chemical markers for sea salt in IMPROVE aerosol data. *Atmos. Environ.* **42**, 261-274, doi:https://doi.org/10.1016/j.atmosenv.2007.09.040 (2008).
42 Solomon, P. A. *et al.* U.S. National PM2.5 Chemical Speciation Monitoring Networks—CSN and IMPROVE: Description of networks. *Journal of the Air & Waste Management Association* **64**, 1410-1438, doi:10.1080/10962247.2014.956904 (2014).
43 Li, C. *et al.* Trends in Chemical Composition of Global and Regional Population-Weighted Fine Particulate Matter Estimated for 25 Years. *Environ. Sci. Technol.* **51**, 11185-11195, doi:10.1021/acs.est.7b02530 (2017).
44 Xu, Q. *et al.* Nitrate dominates the chemical composition of $PM_{2.5}$ during haze event in Beijing, China. *Science of The Total Environment* **689**, 1293-1303, doi:https://doi.org/10.1016/j.scitotenv.2019.06.294 (2019).
45 Zhao, L. *et al.* Changes of chemical composition and source apportionment of $PM_{2.5}$ during 2013–2017 in urban Handan, China. *Atmos. Environ.* **206**, 119-131, doi:https://doi.org/10.1016/j.atmosenv.2019.02.034 (2019).

46 Huang, F. *et al.* Chemical characteristics and source apportionment of $PM_{2.5}$ in Wuhan, China. *Journal of Atmospheric Chemistry* **76**, 245-262, doi:10.1007/s10874-019-09395-0 (2019).
47 Zhang, H.-T. *et al.* [Spatial Temporal Characteristics and Cluster Analysis of Chemical Components for Ambient PM2.5 in Wuhan]. *Huan Jing Ke Xue* **40**, 4764-4773, doi:10.13227/j.hjkx.201904069 (2019).
48 Zhang, Q. *et al.* Drivers of improved $PM_{2.5}$ air quality in China from 2013 to 2017. *P. Natl. Acad. Sci.* **116**, 24463, doi:10.1073/pnas.1907956116 (2019).
49 McNeill, J. *et al.* Large global variations in measured airborne metal concentrations driven by anthropogenic sources. *Scientific Reports* **10**, 21817, doi:10.1038/s41598-020-78789-y (2020).
50 Pandey, A., Shetty, N. J. & Chakrabarty, R. K. Aerosol light absorption from optical measurements of PTFE membrane filter samples: sensitivity analysis of optical depth measures. *Atmos. Meas. Tech.* **12**, 1365-1373, doi:10.5194/amt-12-1365-2019 (2019).
51 Dillner, A. M. & Takahama, S. Predicting ambient aerosol thermal-optical reflectance (TOR) measurements from infrared spectra: organic carbon. *Atmos. Meas. Tech.* **8**, 1097-1109, doi:10.5194/amt-8-1097-2015 (2015).
52 O'Brien, R. E. *et al.* Ultrasonic nebulization for the elemental analysis of microgram-level samples with offline aerosol mass spectrometry. *Atmos. Meas. Tech.* **12**, 1659-1671, doi:10.5194/amt-12-1659-2019 (2019).
53 Hoesly, R. M. *et al.* Historical (1750–2014) anthropogenic emissions of reactive gases and aerosols from the Community Emissions Data System (CEDS). *Geosci. Model Dev.* **11**, 369-408, doi:10.5194/gmd-11-369-2018 (2018).
54 McDuffie, E. E. *et al.* A global anthropogenic emission inventory of atmospheric pollutants from sector- and fuel-specific sources (1970–2017): an application of the Community Emissions Data System (CEDS). *Earth Syst. Sci. Data* **12**, 3413-3442, doi:10.5194/essd-12-3413-2020 (2020).
55 McDuffie, E. E. *et al.* *CEDS_GBD-MAPS_SourceCode_2020_v1.0*, 2020).
56 McDuffie, E. E. *et al.* CEDS_GBD-MAPS: Global Anthropogenic Emission Inventory of NOx, SO2, CO, NH3, NMVOCs, BC, and OC from 1970-2017 (Version 2020_v1.0). doi:https://doi.org/10.5281/zenodo.3754964 (2020).
57 Crippa, M. *et al.* Gridded emissions of air pollutants for the period 1970–2012 within EDGAR v4.3.2. *Earth Syst. Sci. Data* **10**, 1987-2013, doi:10.5194/essd-10-1987-2018 (2018).
58 Amann, M. *et al.* Cost-effective control of air quality and greenhouse gases in Europe: Modeling and policy applications. *Environmental Modelling & Software* **26**, 1489-1501, doi:https://doi.org/10.1016/j.envsoft.2011.07.012 (2011).
59 Klimont, Z. *et al.* Global anthropogenic emissions of particulate matter including black carbon. *Atmos. Chem. Phys.* **17**, 8681-8723, doi:10.5194/acp-17-8681-2017 (2017).
60 Keller, C. A. *et al.* HEMCO v1.0: a versatile, ESMF-compliant component for calculating emissions in atmospheric models. *Geosci. Model Dev.* **7**, 1409-1417, doi:10.5194/gmd-7-1409-2014 (2014).
61 Travis, K. R. *et al.* Why do models overestimate surface ozone in the Southeast United States? *Atmos. Chem. Phys.* **16**, 13561-13577, doi:10.5194/acp-16-13561-2016 (2016).

62 Vinken, G. C. M., Boersma, K. F., Jacob, D. J. & Meijer, E. W. Accounting for non-linear chemistry of ship plumes in the GEOS-Chem global chemistry transport model. *Atmos. Chem. Phys.* **11**, 11707-11722, doi:10.5194/acp-11-11707-2011 (2011).
63 Holmes, C. D., Prather, M. J. & Vinken, G. C. M. The climate impact of ship NO_x emissions: an improved estimate accounting for plume chemistry. *Atmos. Chem. Phys.* **14**, 6801-6812, doi:10.5194/acp-14-6801-2014 (2014).
64 van der Werf, G. R. *et al.* Global fire emissions estimates during 1997–2016. *Earth Syst. Sci. Data* **9**, 697-720, doi:10.5194/essd-9-697-2017 (2017).
65 Mu, M. *et al.* Daily and 3-hourly variability in global fire emissions and consequences for atmospheric model predictions of carbon monoxide. *J. Geophys. Res. Atmos.* **116**, doi:10.1029/2011JD016245 (2011).
66 Philip, S. *et al.* Anthropogenic fugitive, combustion and industrial dust is a significant, underrepresented fine particulate matter source in global atmospheric models. *Environmental Research Letters* **12**, 044018, doi:10.1088/1748-9326/aa65a4 (2017).
67 Fairlie, D. T., Jacob, D. J. & Park, R. J. The impact of transpacific transport of mineral dust in the United States. *Atmos. Environ.* **41**, 1251-1266, doi:https://doi.org/10.1016/j.atmosenv.2006.09.048 (2007).
68 Fairlie, T. D. *et al.* Impact of mineral dust on nitrate, sulfate, and ozone in transpacific Asian pollution plumes. *Atmos. Chem. Phys.* **10**, 3999-4012, doi:10.5194/acp-10-3999-2010 (2010).
69 Stettler, M. E. J., Eastham, S. & Barrett, S. R. H. Air quality and public health impacts of UK airports. Part I: Emissions. *Atmos. Environ.* **45**, 5415-5424, doi:https://doi.org/10.1016/j.atmosenv.2011.07.012 (2011).
70 Murray, L. T., Jacob, D. J., Logan, J. A., Hudman, R. C. & Koshak, W. J. Optimized regional and interannual variability of lightning in a global chemical transport model constrained by LIS/OTD satellite data. *J. Geophys. Res. Atmos.* **117**, doi:10.1029/2012JD017934 (2012).
71 Hudman, R. C. *et al.* Steps towards a mechanistic model of global soil nitric oxide emissions: implementation and space based-constraints. *Atmos. Chem. Phys.* **12**, 7779-7795, doi:10.5194/acp-12-7779-2012 (2012).
72 Fischer, E. V., Jacob, D. J., Millet, D. B., Yantosca, R. M. & Mao, J. The role of the ocean in the global atmospheric budget of acetone. *Geophysical Research Letters* **39**, doi:10.1029/2011GL050086 (2012).
73 Millet, D. B. *et al.* Global atmospheric budget of acetaldehyde: 3-D model analysis and constraints from in-situ and satellite observations. *Atmos. Chem. Phys.* **10**, 3405-3425, doi:10.5194/acp-10-3405-2010 (2010).
74 Breider, T. J. *et al.* Multidecadal trends in aerosol radiative forcing over the Arctic: Contribution of changes in anthropogenic aerosol to Arctic warming since 1980. *J. Geophys. Res. Atmos.* **122**, 3573-3594, doi:10.1002/2016JD025321 (2017).
75 Riddick, S. N. *et al.* Global ammonia emissions from seabirds (NERC Environmental Information Data Centre). doi:https://doi.org/10.5285/c9e802b3-43c8-4b36-a3a3-8861d9da8ea9 (2012).
76 Croft, B. *et al.* Contribution of Arctic seabird-colony ammonia to atmospheric particles and cloud-albedo radiative effect. *Nature Communications* **7**, 13444, doi:10.1038/ncomms13444 https://www.nature.com/articles/ncomms13444#supplementary-information (2016).

77 Carpenter, L. J. *et al.* Atmospheric iodine levels influenced by sea surface emissions of inorganic iodine. *Nature Geoscience* **6**, 108-111, doi:10.1038/ngeo1687 (2013).
78 Guenther, A. B. *et al.* The Model of Emissions of Gases and Aerosols from Nature version 2.1 (MEGAN2.1): an extended and updated framework for modeling biogenic emissions. *Geosci. Model Dev.* **5**, 1471-1492, doi:10.5194/gmd-5-1471-2012 (2012).
79 Liang, Q. *et al.* Finding the missing stratospheric Br_y: a global modeling study of $CHBr_3$ and CH_2Br_2. *Atmos. Chem. Phys.* **10**, 2269-2286, doi:10.5194/acp-10-2269-2010 (2010).
80 Ordóñez, C. *et al.* Bromine and iodine chemistry in a global chemistry-climate model: description and evaluation of very short-lived oceanic sources. *Atmos. Chem. Phys.* **12**, 1423-1447, doi:10.5194/acp-12-1423-2012 (2012).
81 Lelieveld, J. *et al.* Effects of fossil fuel and total anthropogenic emission removal on public health and climate. *P. Natl. Acad. Sci.* **116**, 7192, doi:10.1073/pnas.1819989116 (2019).
82 Marais, E. A. *et al.* Air Quality and Health Impact of Future Fossil Fuel Use for Electricity Generation and Transport in Africa. *Environ. Sci. Technol.* **53**, 13524-13534, doi:10.1021/acs.est.9b04958 (2019).
83 Lacey, F. G. *et al.* Improving present day and future estimates of anthropogenic sectoral emissions and the resulting air quality impacts in Africa. *Faraday Discussions* **200**, 397-412, doi:10.1039/C7FD00011A (2017).
84 Chafe, Z. A. *et al.* Household Cooking with Solid Fuels Contributes to Ambient PM2.5 Air Pollution and the Burden of Disease. *Environmental Health Perspectives* **122**, 1314-1320, doi:10.1289/ehp.1206340 (2014).
85 Wu, R. *et al.* Air quality and health benefits of China's emission control policies on coal-fired power plants during 2005–2020. *Environmental Research Letters* **14**, 094016, doi:10.1088/1748-9326/ab3bae (2019).
86 GBD MAPS Working Group. *Burden of Disease Attributable to Coal-Burning and Other Major Sources of Air Pollution in China. Special Report 20*. (Health Effects Institute [Available at: https://www.healtheffects.org/publication/burden-disease-attributable-coal-burning-and-other-air-pollution-sources-china], 2016).
87 GBD MAPS Working Group. *Burden of Disease Attributable to Major Air Pollution Sources in India. Special Report 21.*, (Health Effects Institute [Available at: https://www.healtheffects.org/publication/gbd-air-pollution-india], 2018).
88 Zheng, B. *et al.* Trends in China's anthropogenic emissions since 2010 as the consequence of clean air actions. *Atmos. Chem. Phys.* **18**, 14095-14111, doi:10.5194/acp-18-14095-2018 (2018).
89 Zhang, Z. *et al.* The contribution of residential coal combustion to PM2.5 pollution over China's Beijing-Tianjin-Hebei region in winter. *Atmos. Environ.* **159**, 147-161, doi:https://doi.org/10.1016/j.atmosenv.2017.03.054 (2017).
90 Liu, J. *et al.* Air pollutant emissions from Chinese households: A major and underappreciated ambient pollution source. *P. Natl. Acad. Sci.* **113**, 7756, doi:10.1073/pnas.1604537113 (2016).
91 Lelieveld, J., Evans, J. S., Fnais, M., Giannadaki, D. & Pozzer, A. The contribution of outdoor air pollution sources to premature mortality on a global scale. *Nature* **525**, 367, doi:10.1038/nature15371 (2015).
92 Gu, Y. *et al.* Impacts of sectoral emissions in China and the implications: air quality, public health, crop production, and economic costs. *Environmental Research Letters* **13**, 084008, doi:10.1088/1748-9326/aad138 (2018).

93 Hu, J. *et al.* Premature Mortality Attributable to Particulate Matter in China: Source Contributions and Responses to Reductions. *Environ. Sci. Technol.* **51**, 9950-9959, doi:10.1021/acs.est.7b03193 (2017).
94 Meng, J. *et al.* Source Contributions to Ambient Fine Particulate Matter for Canada. *Environ. Sci. Technol.* **53**, 10269-10278, doi:10.1021/acs.est.9b02461 (2019).
95 Caiazzo, F., Ashok, A., Waitz, I. A., Yim, S. H. L. & Barrett, S. R. H. Air pollution and early deaths in the United States. Part I: Quantifying the impact of major sectors in 2005. *Atmos. Environ.* **79**, 198-208, doi:https://doi.org/10.1016/j.atmosenv.2013.05.081 (2013).
96 Fann, N., Fulcher, C. M. & Baker, K. The Recent and Future Health Burden of Air Pollution Apportioned Across U.S. Sectors. *Environ. Sci. Technol.* **47**, 3580-3589, doi:10.1021/es304831q (2013).
97 Penn Stefani, L. *et al.* Estimating State-Specific Contributions to PM2.5- and O3-Related Health Burden from Residential Combustion and Electricity Generating Unit Emissions in the United States. *Environmental Health Perspectives* **125**, 324-332, doi:10.1289/EHP550 (2017).
98 Thakrar, S. K. *et al.* Reducing Mortality from Air Pollution in the United States by Targeting Specific Emission Sources. *Environ. Sci. Tech. Let.*, doi:10.1021/acs.estlett.0c00424 (2020).
99 Bond, T. C. *et al.* A technology-based global inventory of black and organic carbon emissions from combustion. *J. Geophys. Res. Atmos.* **109**, doi:10.1029/2003JD003697 (2004).
100 Butt, E. W. *et al.* The impact of residential combustion emissions on atmospheric aerosol, human health, and climate. *Atmos. Chem. Phys.* **16**, 873-905, doi:10.5194/acp-16-873-2016 (2016).
101 Crippa, M., Janssens-Maenhout, G., Guizzardi, D., Van Dingenen, R. & Dentener, F. Contribution and uncertainty of sectorial and regional emissions to regional and global PM2.5 health impacts. *Atmos. Chem. Phys.* **19**, 5165-5186, doi:10.5194/acp-19-5165-2019 (2019).
102 Giannadaki, D., Pozzer, A. & Lelieveld, J. Modeled global effects of airborne desert dust on air quality and premature mortality. *Atmos. Chem. Phys.* **14**, 957-968, doi:10.5194/acp-14-957-2014 (2014).
103 Pozzer, A., Tsimpidi, A. P., Karydis, V. A., de Meij, A. & Lelieveld, J. Impact of agricultural emission reductions on fine-particulate matter and public health. *Atmos. Chem. Phys.* **17**, 12813-12826, doi:10.5194/acp-17-12813-2017 (2017).
104 Guo, H. *et al.* Effectiveness of ammonia reduction on control of fine particle nitrate. *Atmos. Chem. Phys.* **18**, 12241-12256, doi:10.5194/acp-18-12241-2018 (2018).
105 Anenberg, S., Miller, J., Henze, D. & Minjares, R. A global snapshot of the air pollution-related health impacts of transportation sector emissions in 2010 and 2015. *The International Council on Clean Transportation (ICCT). [Available at: https://theicct.org/publications/health-impacts-transport-emissions-2010-2015]* (2019).
106 Chambliss, S. E., Silva, R., West, J. J., Zeinali, M. & Minjares, R. Estimating source-attributable health impacts of ambient fine particulate matter exposure: global premature mortality from surface transportation emissions in 2005. *Environmental Research Letters* **9**, 104009, doi:10.1088/1748-9326/9/10/104009 (2014).
107 Silva Raquel, A., Adelman, Z., Fry Meridith, M. & West, J. J. The Impact of Individual Anthropogenic Emissions Sectors on the Global Burden of Human Mortality due to Ambient

Air Pollution. *Environmental Health Perspectives* **124**, 1776-1784, doi:10.1289/EHP177 (2016).
108 Tsimpidi, A. P., Karydis, V. A. & Pandis, S. N. Response of Fine Particulate Matter to Emission Changes of Oxides of Nitrogen and Anthropogenic Volatile Organic Compounds in the Eastern United States. *Journal of the Air & Waste Management Association* **58**, 1463-1473, doi:10.3155/1047-3289.58.11.1463 (2008).
109 McDonald, B. C. *et al.* Volatile chemical products emerging as largest petrochemical source of urban organic emissions. *Science* **359**, 760, doi:10.1126/science.aaq0524 (2018).
110 Corbett, J. J. *et al.* Mortality from Ship Emissions: A Global Assessment. *Environ. Sci. Technol.* **41**, 8512-8518, doi:10.1021/es071686z (2007).
111 Sofiev, M. *et al.* Cleaner fuels for ships provide public health benefits with climate tradeoffs. *Nature Communications* **9**, 406, doi:10.1038/s41467-017-02774-9 (2018).
