# Supplementary material for: Source sector and fuel contributions to ambient PM2.5 and attributable mortality across multiple spatial scales
Source: Nat Commun. 2021 Jun 14;12:3594. doi: 10.1038/s41467-021-23853-y (PMC8203641; doi:10.1038/s41467-021-23853-y)
Supplement: Supplementary file 3 — Supplementary Data File Descriptions [file 41467_2021_23853_MOESM3_ESM.pdf]

Description of Additional Supplementary Data Files

File Name: Supplementary Data 1
Description: Global, regional, national, and sub-national $PM_{2.5}$ exposure estimates and sector and disease-specific fractional contributions. Provides downscaled population weighted mean (PWM) national-level $PM_{2.5}$ exposure estimates for 200 sub-national areas, 204 countries and territories, and 21 world regions. This table also provides the total attributable deaths and the number of neonatal incidences associated with PWM $PM_{2.5}$ exposure levels in each country and region. Burden results are provided from both the 2019 Global Burden of Disease (GBD2019) concentration response relationships and the Global Exposure Mortality Model (GEMM). Also includes the fractional contributions (units of percent) of each source sector and disease to the total GBD2019 and GEMM disease burden estimates.

File Name: Supplementary Data 2
Description: Global, regional, national, and sub-national $PM_{2.5}$ exposure estimates and combustion fuel-type fractional contributions. Includes relative fuel-type contributions from the combustion of coal, solid biofuel, and the sum of oil and gas.

File Name: Supplementary Data 3
Description: Global, regional, national, and sub-national $PM_{2.5}$ exposure estimates for the year 2019.
